# Perceptions of persons who wear face coverings are modulated by the perceivers’ attitude

**DOI:** 10.3389/fnins.2022.988546

**Published:** 2022-11-04

**Authors:** Johannes Leder, Lisa Koßmann, Claus-Christian Carbon

**Affiliations:** ^1^Institute of Psychology, University of Bamberg, Bamberg, Germany; ^2^Bamberg Graduate School of Affective and Cognitive Sciences (BaGrACS), Bamberg, Germany

**Keywords:** head cover, facial mask, COVID-19, attractiveness, prosociality, social attitude, theory of mind, hijab

## Abstract

We examined if the effect of facial coverings on person perception is influenced by the perceiver’s attitudes. We used two online experiments in which participants saw the same human target persons repeatedly appearing with and without a specific piece of clothing and had to judge the target persons’ character. In Experiment 1 (*N* = 101), we investigated how the wearing of a facial mask influences a person’s perception depending on the perceiver’s attitude toward measures against the COVID-19 pandemic. In Experiment 2 (*N* = 114), we examined the effect of wearing a head cover associated with Arabic culture on a person’s perception depending on the perceiver’s attitude toward Islam. Both studies were preregistered; both found evidence that a person’s perception is a process shaped by the personal attitudes of the perceiver as well as merely the target person’s outward appearance. Integrating previous findings, we demonstrate that facial covers, as well as head covers, operate as cues which are used by the perceivers to infer the target persons’ underlying attitudes. The judgment of the target person is shaped by the perceived attitude toward what the facial covering stereotypically symbolizes.

## Introduction

Perceived attractiveness, liking, and character judgments of people wearing facial covering are strongly influenced by the attitude of the perceiver.

Humans infer on characteristics of others based on visible cues. The judgments of attractiveness (Carbon et al., 2010), liking, and personal character are based on visual cues, particularly in faces (Willis and Todorov, 2006; Todorov et al., 2014). While cues seem to be universal for attractiveness as rater agreement measured is high between different perceivers (Langlois et al., 2000), the judgment of attractiveness is fueled by personal taste and shared taste, suggesting that cues are interpreted to equal parts based on commonly held assumptions and uniquely held assumptions (Hönekopp, 2006). The similarity of facial features with personally relevant others fuels the personal component of judgment (Kraus and Chen, 2010; Günaydin et al., 2012). Stereotypes are shared social knowledge regarding specific groups, which result in person judgments (Maddox, 2004) and can even influence behavior such as trusting (Stanley et al., 2011) or concrete social behavior such as helpfulness (see Wheeler and Petty, 2001).

Humans do not rely on faces alone when judging their counterparts but also use other cues. These cues can stem from a wide range of visible properties, for example, a person’s clothing to infer their self-concept (Piacentini and Mailer, 2004), a person’s hair to infer their ideology (Synnott, 1987), or religious paraphernalia to assess a person’s religion and cultural heritage (Taylor et al., 2010; Desai and Kouchaki, 2017).

These findings point to the effect of top-down information processing during person perception. Cues activate certain schemata that are associated with specific characterizations, which in turn are applied to the specific person. This fast initial assessment carries over onto subsequent appraisals and behavior toward the object of assessment as the first impression is stable over time (Willis and Todorov, 2006).

The outside appearances of people matter, because the top-down judgments guided by these visible cues have far-reaching consequences. Meta-analyses show consistently that the skin color of the judged person influences the jury’s decisions concerning convictions (Mitchell et al., 2005) and workplace decisions (Koch et al., 2015), and a similar effect is observed for attractiveness (Mazzella and Feingold, 1994). Importantly, in the case of juror decisions, these effects seem to be moderated by characteristics on the perceiver side (Devine and Caughlin, 2014). But do perceiver characteristics in general shape the perception of others depending on the visual cues they have? Further factors seem to matter, most importantly, visual perception of social categories is also known to be shaped by higher order social cognitive processes. If we show negative attitudes, possess stereotypes about certain groups of persons or if we follow specific goals, our visual perception is biased (Freeman and Johnson, 2016), which can lead to very unfortunate, e.g., racial biases (Harsányi and Carbon, 2015; Bagnis et al., 2020). Understanding such mechanisms involved in making initial (and sometimes persistent) judgments is crucial in understanding human interactions.

Previous research has shown that top-down information processing is induced by cues, which then shape a person’s perception (Carbon et al., 2018). Wearing a mask by a target person is a specific cue for underlying attitudes, importantly, person perception is not only a function of the attitudes of the target person but also of the perceivers’ attitudes—we like people who are similar to us (Byrne and Griffitt, 1973). Attraction to strangers and perceived similarity correlate with *r* = 0.49 according to a meta-analysis (Montoya et al., 2008). Importantly, the studies investigating the effect of attitude similiarity between the perceived and the perceiver on person judgments explicitly present the attitude of the stranger to participants (e.g., Pilkington and Lydon, 1997; Singh et al., 2007). Facial coverings in many ways are a signal of specific attitudes held by the wearer. A person wearing a Kippah is potentially expressing Jewish faith, and persons wearing MAGA caps aim to express their support for Donald Trump. Are these facial coverings affecting how the person wearing them is perceived, depending on the perceiver’s attitudes?

In the present study, two cues are examined, which are imperative to be understood particularly in today’s political and social climates: medical face-coverings and Arabic headdresses. We aim to show that cues are not judged equally but their implications for characterization are dependent on the perceiver.

## The present research

To what degree does the judgment of another person depend on the perceiver’s attitude toward an issue associated with the visible cue, that is, its’ symbolic value, but not the person itself? Investigating the effect of cues and attitudes held by the perceiver and their combined effect on person judgments—racial prejudice has been a prominent example. However, studies focused on racial prejudice and its effect on person perception (e.g., Blair, 2002; Maddox and Gray, 2002; Hugenberg and Sacco, 2008; Quinn and Macrae, 2011) cannot disentangle the effect of the target person and the target person’s appearance linked to the symbol (skin color). Studies interested in the effects of symbols added to the person, such as status symbols, show that the effect of these symbols differs depending on the perceiver (friends vs. strangers) (Garcia et al., 2019). However, here the influence of personal attitudes of the perceiver was not examined—but considering research on stereotypes, the attitude toward the group or issue, the symbol stands for, should explain the shift in judgment. The values of certain symbols are in the eye of the beholder: wearing a mask against COVID-19 could be such a symbol and wearing a *hijab* in females or a *kufiya* in males could be another. We were interested in the effect of these two facial coverings and the perceiver’s attitudes toward issues associated with these facial coverings on person perception and character judgments.

## Experiment 1

**FIGURE 1:**
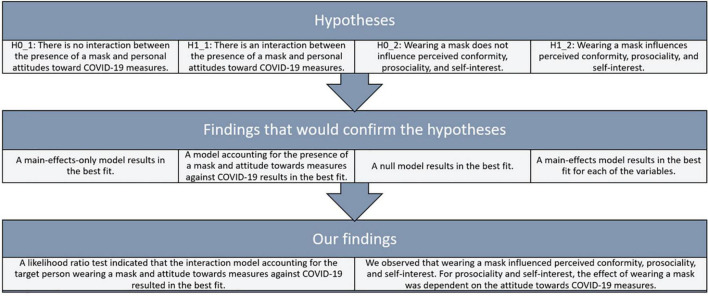
Hypotheses, predicted observations, and findings for experiment 1.

Does the attitude toward measures against COVID-19 influence the perception of a target person wearing masks or no masks regarding the target person’s attractiveness, liking, and character? Previous research shows that wearing a mask results in more positive judgments for some samples (Oldmeadow and Koch, 2021; Hies and Lewis, 2022) but more negative judgments for others (Miyazaki and Kawahara, 2016). Based on our theorizing, we assume that this difference is explained by the underlying differences in attitudes of the perceivers. In an online experiment, we asked participants to repeatedly rate a target person shown in a public place regarding the target person’s character. We varied whether the person wore a facial mask or not to test our two main hypotheses: First, with an increasingly positive attitude toward measures against COVID-19, a person wearing a mask is evaluated more positively on the dimensions of attractiveness and liking than without a mask. Conversely, a target person without a mask is evaluated more positively with decreasing positive attitude toward measures against COVID-19. Second, a target person’s conformity is judged higher with an increasingly critical view of measures against COVID-19, a target person’s prosociality is judged lower with increasing critical view of measures against COVID-19, and a target person’s self-interest is judged lower with increasing critical view of measures against COVID-19. For Experiment 1, hypotheses and findings are summarised in Figure 1.

## Materials and methods

### Sample and design

All materials and the preregistration, registered before data collection, are available at https://osf.io/xqmpw?view_only=64cbd820d23f4bc7b3d84b396ae6c8e4.

The relevant hypothesis for the power analysis, which determined the sample size, was Hypothesis 1 (H1). H1 is an interaction of mask (yes vs. no) and personal attitude toward COVID-19 measures (continuous). We tested two interaction effects, one for liking and one for attractiveness. We used R package {simR} (Green and Macleod, 2016) for the power calculation on basis of a random-effects model accounting for the employed repeated measures design. Detailed model assumptions are explained in the preregistration file. The effect in question is the fixed effect of the interaction between the attitude toward measures against COVID-19 with wearing a mask or not and was set to β = 0.15, given that *r* and β are equivalent when predictors are independent [(Peterson and Brown, 2005) this represents a small effect]. To observe that this effect explains a significant amount of variance compared to the main effects only model with α = 0.01 and a satisfactory test power 1 − β of 0.80 we collected data from *N* = 101 participants (*M*_*age*_ = 35.9 years, 75 women, 23 men, and 1 participant assigned to the “other” category; see Supplementary Table 1 in Electronic supplement A).

Participants’ attitude toward measures against COVID-19 was assessed. Participants were then asked to rate two people depicted under varying conditions, which resulted in an orthogonal within-participants design of the varying factors mask (yes vs. no), target person (male vs. female), and partner (both appear the same way vs. target person differs from other).

### Material

#### Attitude toward measures against COVID-19

The participants’ attitude toward measures against COVID-19 was measured with a self-constructed scale consisting of seven items and their order was randomized before the study. All items are listed in Table 1. Participants responded to each item on a five-point scale (1 = *strong disagreement [starke Ablehnung]*, 2 = *disagreement [Ablehnung]*, 3 = *neutral [neutral]*, 4 = *agreement [Zustimmung]*, 5 = *strong agreement [starke Zustimmung]—*original German terms in brackets).

**TABLE 1:** Items for attitude toward measures against COVID-19 (original German terms in brackets).

No.	Item
(1)	(−) The protective measures are very stressful for me. [Die Schutzmaßnahmen sind für mich sehr belastend.]
(2)	(−) I feel that my freedom is severely restricted by the government’s measures. [Ich fühle mich in meinen Freiheiten stark eingeschränkt durch die Maßnahmen der Regierung.]
(3)	(−) I think government measures, such as Contact restrictions are excessive. [Ich denke, die Maßnahmen der Regierung, wie z.B. Kontaktbeschränkungen, sind überzogen.]
(4)	(−) I feel economically very threatened by the measures against COVID-19 [Ich fühle mich wirtschaftlich sehr bedroht durch Maßnahmen gegen COVID-19.]
(5)	(−) I think COVID-19 is no worse than influenza. [Ich denke COVID-19 ist nicht schlimmer als eine Influenza.]
(6)	I find protective masks a very good way to protect yourself and others from COVID-19. [Ich finde Schutzmasken eine sehr gute Möglichkeit sich und andere vor COVID-19 zu schützen.]
(7)	I feel very threatened by COVID-19. [Ich fühle mich gesundheitlich sehr bedroht durch COVID-19.]

(−) indicate reversed items.

The scale reflected each participant’s mean score and showed satisfactory consistency expressed by a Cronbach’s α = 0.77, *M* = 3.4, and *SD* = 0.77.

#### Stimuli

**FIGURE 2:**
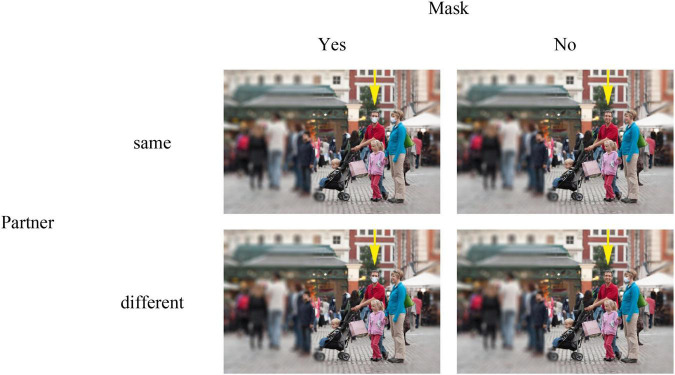
Stimuli used in experiment 1. The figure shows all variations for the male target person. For the female target person, the arrow was moved above the female, respectively. To ensure the anonymity of the people displayed in the photograph, faces were blurred for the published manuscript. The original photo (without face masks and blurring effects) was kindly made public by Jason Pier through a CC BY-NC 2.0 license.

The photograph used as a base stimulus showed a family consisting of two adults and two children. The picture was edited in three ways to avoid confounding. First, the background was blurred so that only the family was in focus and no specifics about the general wearing of masks of other persons were provided—still the picture made the impression that the small family was in the middle of a frequented market square. Second, the respective mask was added or taken away in the same picture. Third, a yellow arrow was added above the target person to indicate who was to be judged by the participant (see Figure 2).

#### Dependent variables

The study was carried out in German, but the items in Table 2 are presented in English here. All items were measured on a 7-point rating scale (1 = *fully disagree [trifft gar nicht zu]*, 7 = *fully agree [trifft vollkommen zu]*).

**TABLE 2:** Items measuring how the participant perceives the target person (the original wording in German is given in brackets).

No	Item
(1)	The person wants to avoid being noticed negatively [Die Person will vermeiden negativ aufzufallen]
(2)	The person wants to be socially accepted [Die Person will sozial akzeptiert sein]
(3)	The person simply follows the government’s recent recommendations [Die Person folgt einfach den jüngsten Empfehlungen der Regierung]
(4)	The person wants to protect others [Die Person will andere schützen]
(5)	The person thinks s/he may be ill and wants to protect other people from infection [Die Person denkt, sie könnte erkrankt sein und will andere Menschen vor einer Ansteckung schützen]
(6)	The person is very prosocial [Die Person ist sehr prosozial]
(7)	The person thinks primarily of herself/himself and does not want to be infected by others, although the probability of this is very low [Die Person denkt primär an sich selbst und will nicht infiziert werden von anderen, obwohl die Wahrscheinlichkeit davon sehr gering ist]
(8)	The person wants to protect himself [Die Person will sich schützen]
(9)	The person is afraid [Die Person hat Angst]
(10)	The person has the coronavirus [Die Person hat das Coronavirus]
(11)	The person is much more scared than s/he should be [Die Person hat viel mehr Angst als sie haben sollte]
(12)	The person looks strange [Die Person sieht eigenartig aus]
(13)	The person is careful [Die Person ist umsichtig]
(14)	The person is neurotic [Die Person ist neurotisch]
(15)	The person is aggressive [Die Person ist aggressiv]
(16)	The person is attractive [Die Person ist attraktiv]
(17)	The person is liked by me [Die Person ist sympathisch]

Here the English translation is shown. Original German wording in brackets. The two main dependent variables were attractiveness and liking—they were both measured by one single item each. Perceived attractiveness was measured with Item #16, and liking was assessed with Item #17.

The measure of three characteristics of the target persons, perceived conformity, perceived prosociality, and perceived self-interest consisted of three items each. Items #1–#9 were aggregated to three scales reflecting *Conformity* (Items #1–#3), *Prosociality* (Items #4–#6), and *Self-interest* (Items #7–#9). We conducted a multilevel reliability analysis with the package {psych} (Revelle, 2019). The reliability for each scale, conformity, prosociality, and self-interest, was estimated based on Formula #11 given by Shrout and Lane (2013). This formula estimates the reliability of between-person differences, averaged over items. The resulting coefficient is referred to as *Rcn*. The respective *Rcn* for conformity was 0.62, for prosociality was 0.70, and for self-interest was 0.61.

### Procedure

Between 29 May 2020 and 25 June 2020, participants were invited to an online study through different recruitment tools, mainly a university-specific one and a mailing list using ORSEE (Greiner, 2015). Furthermore, participants’ attitudes toward measures against COVID-19 were assessed. Then, participants viewed eight consecutive pictures showing a small family (consisting of a mother, a father, a female child at kindergarten age, and a male child at toddler age sitting in a baby buggy) standing in a public place. The order of pictures was randomized for each participant. Participants were asked to rate the target person in the picture (the target person was indicated by a vertical arrow from above, directed toward the person’s head; for an illustration of typical stimuli, see Figure 2). The ratings captured liking, attractiveness, perceived prosociality, perceived conformity, and perceived self-interest of the target person. The setting of the pictures was identical, with the exception that we systematically manipulated whether the adults wore a mask or not with all combinations being available (female/male: no mask/no mask, no mask/mask, mask/no mask, and mask/mask), we further manipulated which adult we indicated as target person (mother vs. father). After completion of the eight trials, one picture was randomly selected and presented to a participant, who was asked to provide a written description of the scene including what they thought was on the mind of the depicted people. This measure was not relevant to the current study. Finally, participants responded to the questions about demographics and were thanked for their participation.

### Statistical analysis

We used linear multilevel regressions with participants’ ID as a random effect to account for the repeated measures. For the analysis, we mean-centered the variable attitude toward measures against COVID-19; all factorial variables were dummy coded. For executing the multilevel linear analyses, we used the R package {lmer} (Bates et al., 2015). For the analysis of slopes, we used the R package {interactions} (Long, 2019). Because treating ordinal responses as continuous can result in wrong inferences (Liddell and Kruschke, 2018), we also report results based on an ordinal regression in a Bayesian fashion (see Electronic supplement F).

## Results

### Test of preregistered hypothesis H1

**FIGURE 3:**
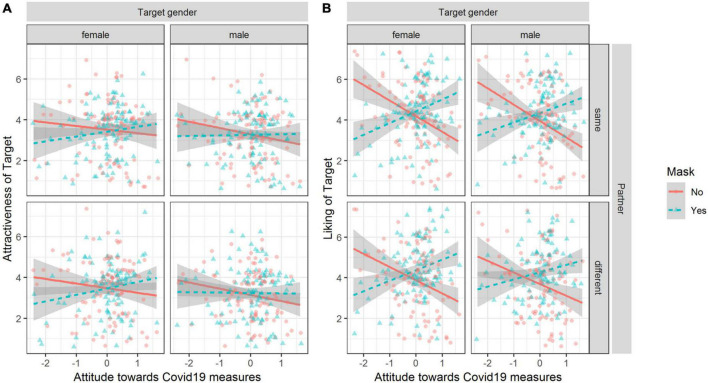
Observed attractiveness and liking ratings dependent on attitude, mask, target person gender, and partner. Plot **(A)** for attractiveness. Plot **(B)** for liking. Lines depict linear regression of Y on X and the shaded area shows the 95% CI of the estimate. Data points are jittered to avoid overlap.

We observed that perceived attractiveness and liking were dependent on the participants’ attitude toward measures against COVID-19 and whether the target person was wearing a mask or not (see Figure 3). We fit regression models for attractiveness and liking individually (for all estimates, see Supplementary Table 2 in the Electronic supplement).

A likelihood ratio test indicated that the interaction model accounting for the target person wearing a mask and attitude toward measures against COVID-19 resulted in the best fit compared to the main effects only model, χ^2^ (1) = 23.17, *p* < 0.001—fixed effects explained 3% of the variance (marg. *R*^2^ = 0.03). Adding the other interactions did not significantly improve the overall fit. The effect of wearing a mask compared to not wearing a mask on attractiveness, β = 0.00, 95% CI [−0.05, 0.04], was moderated by the attitude toward measures against COVID-19, β = 0.12, 95% CI [0.07, 0.17]. To test in which regions the slopes differed, we used the Johnson-Neyman procedure which estimates the region of a significant difference between the slopes. The region of a significant difference of slopes depending on the moderator was [−0.46, +1.65] and participants’ who had attitudes outside below/above these boundaries had lower/higher judgment about the attractiveness of a target person with a mask compared to the same target person without a mask.

For liking, we observed the same pattern of results. The interaction model including the interaction of mask and attitudes toward measures against COVID-19 resulted in a better fit compared to the main effects only model, χ^2^ (1) = 127.33, *p* < 0.001; fixed effects explained 13% of the variance (marg. *R*^2^ = 0.13). Adding the other interactions did not significantly increase fit. The effect of wearing a mask compared to not wearing a mask on liking, β = 0.14, 95% CI [0.09, 0.20], was moderated by the attitude toward measures against COVID-19, β = 0.31, 95% CI [0.26, 0.36].

The region of significant difference of slopes depending on the moderator was [−0.45, 0.04] and participants who have attitudes below/above these boundaries expressed less/more liking when comparing a person with a mask to the same person without a mask. We obtained the same significant interaction effects supporting H1 when using a Bayesian ordered-probit regression (see Electronic supplement C).

### Test of preregistered hypothesis H2

**FIGURE 4:**
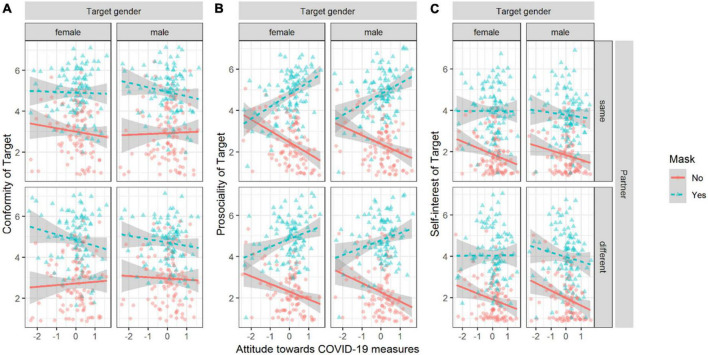
Perceived judgment of the target person depending on mask, partner, and gender of target person. Plot **(A)** for perceived conformity, plot **(B)** for perceived prosociality, and Plot **(C)** for self-interest. Lines depict linear regression of Y on X; the shaded area indicates the 95% CI of the estimate. Points are jittered to avoid overlap.

We observed that wearing a mask influenced perceived conformity, prosociality, and self-interest. For prosociality and self-interest, the effect of wearing a mask was dependent on the attitude toward COVID-19 measures (see Figure 4).

We fit three linear regression models for conformity, prosociality, and self-interest. Conformity was dependent on wearing a mask or not (for all estimates, see Supplementary Table 3 in the Electronic supplement). For conformity, the main effects model fit the data best, compared to the null model, χ^2^ (4) = 427.64, *p* < 0.001; fixed effects explained 40% of the variance (marg. *R*^2^ = 0.40). Adding the other interactions did not significantly increase fit. In the full factorial model, conformity was judged higher for target persons wearing a mask compared to target persons not wearing a mask, β = 0.62, 95% CI [0.58, 0.67].

For prosociality, the interaction model, including the interaction of mask and attitudes toward measures against COVID-19, resulted in a better fit compared to the main effects only model, χ^2^ (1) = 127.33, *p* < 0.001, and fixed effects explained 60% of the variance (marg. *R*^2^ = 0.60). Prosociality was judged higher when the target person was wearing a mask compared to not wearing a mask, β = 0.75, 95% CI [0.71, 0.79]. This effect was moderated by the attitude toward measures against COVID-19, β = 0.22, 95% CI [0.18, 0.26]. The region of significant difference of slopes depending on the moderator was [−2.86, −1.54]. Participants who had attitudes below/above these boundaries judged prosociality more negatively/positively when comparing a person with a mask to the same person without a mask.

For self-interest, the interaction model including the interaction of mask and attitudes toward measures against COVID-19 resulted in a better fit compared to the main effects only model, χ^2^ (1) = 6.67, *p* = 0.01, and fixed effects explained 47% of the variance (marg. *R*^2^ = 0.47). Self-interest was judged higher when the target person was wearing a mask compared to not wearing a mask, β = 0.67, 95% CI [0.63, 0.72]. This effect was moderated by the attitude toward measures against COVID-19, β = 0.06, 95% CI [0.01, 0.10]. The region of significant difference of slopes depending on the moderator is inside the interval of [−3.24, 99.41], and none of the observed values fall outside this area (they are restricted to [−3, 3]). The slope of the effect of wearing a mask or not is positive for all observed attitudes toward measures against COVID-19.

### Robustness check

To check the robustness of our results, we used a Bayesian analysis for ordinal linear regression. The results are consistent with our main analysis. Estimates and plots of marginal effects are found in Electronic supplement C.

## Discussion

We observed that the perception of a person wearing a mask or not (the target person) is dependent on the perceiver. The main hypothesis (H1) was an interaction of mask (yes vs. no) and personal attitude toward COVID-19 measures (continuous) for perceived attractiveness as well as for liking of the target person. Our data corroborated H1. Target persons wearing masks are perceived as more attractive and are more liked by people who have strong positive attitudes toward measures against COVID-19, but people who have strong negative attitudes toward measures against COVID-19 do not perceive them as more attractive and do not like them more. For target persons not wearing a mask, the direction of the relationship between attitude and judgment of the target person was reversed. The effects were stronger for liking than for attractiveness. In line with H2, we observed that perceived prosociality and self-interest were the results of an interaction between the target person wearing a mask or not and the perceivers’ attitude toward COVID-19. We did not observe this interaction in the case of conformity judgments.

Wearing masks is a new phenomenon in Western countries and wearing masks or not has direct social consequences—but does the effect observed with masks generalize to the perception of people wearing other symbols which indicate attitudes such as religion? To answer this question, we carried out Experiment 2.

## Experiment 2

**FIGURE 5:**
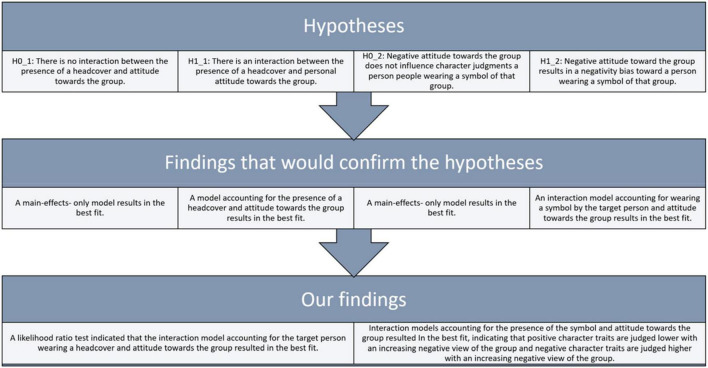
Hypotheses, predicted observations, and findings for experiment 2.

The second study investigated if the effect of mask-wearing is dependent on the attitudes of the perceiver and is a general property of person perception. For this reason, in Experiment 2, we inspected the effect of wearing a hijab for women or wearing a kufiyah for men on person perception. In an online experiment, we asked participants to repeatedly rate a target person shown in a public place regarding the target person’s character. We varied whether the person wore a headscarf or not to address two research questions: First, does the attitude toward the specific group (here: Muslims) influence sympathy toward and perceived attractiveness of people wearing a “symbol” (here: wearing paraphernalia referring to Muslim culture) representative of that group? We hypothesize an interaction of head cover (yes vs. no) and personal attitude toward the group (continuous). The interaction is driven by the simple effect that the person wearing a head cover is judged less attractive and less liked with the perceivers’ increasing negative attitude toward the group. Second, does a negative attitude toward the specific group result in a negative bias toward a person wearing a headcover? We tested the following hypotheses. Positive character traits are judged lower with an increasingly negative view of the group. Negative character traits are judged higher with increasing negative views of the group. For Experiment 2, hypotheses and findings are summarised in Figure 5.

## Method

### Sample and design

All materials and the preregistration, which were submitted before the study was started, are available at https://osf.io/7mnuv?view_only=64cbd820d23f4bc7b3d84b396ae6c8e4.

The relevant hypothesis for the power analysis, which determined the sample size, was Hypothesis 1 (H1). H1 is an interaction of head cover (yes vs. no) and personal attitude toward the group (continuous). We tested two interaction effects, one for liking and one for attractiveness. We used R package {simR} (Green and Macleod, 2016) for the power calculation on the basis of a random-effects model, accounting for the employed repeated measures design. Detailed model assumptions are explicated in the preregistration. The effect in question is the fixed effect of the interaction between the attitude toward Muslims wearing a hijab/kufiyah or not and was set to β = 0.15, which is a small effect. To find that this effect explains a significant amount of variance compared to the main effects only model with α = 0.01 and a satisfactory test power 1 − β of 0.80, we collected data between 18 January 2021 and 1 March 2021 from *N* = 114 (*M*_*age*_ = 35.0 years, 89 female, 24 male, and 1 other, see Supplementary Table 2 in the Electronic supplement D).

Participants’ attitude toward Muslims and Islam was assessed. Participants then were asked to rate a person depicted under varying conditions, which resulted in an orthogonal within-participants design of the varying factors head cover (yes vs. no), target person (male vs. female), and partner (both appear the same way vs. target person differs from other).

### Material

#### Attitude toward group

To assess general attitudes toward foreigners, we used the items of Heitmeyer (2005) assessing attitudes toward foreigners and racism (items 1–4). To measure the specific attitude toward the group (items 5–7), we used the items assessing islamophobia from the “Antidiskriminierungsstelle des Bundes” authorized by the Federal Ministry for Family Affairs, Senior Citizens, Women and Youth (Germany).^1^ The responses were measured through a rating scale of 1 (*do not agree*) – 10 (*totally agree*). Items 4, 5, and 6 were reversed. We coded items so that a higher score reflects a more positive attitude toward the group (all items are listed in Table 3).

**TABLE 3:** Items for attitude toward group.

No	Item
(1)	The whites are rightly leaders in the world. [Die Weißen sind zu Recht führend in der Welt.]
(2)	Too many foreigners live in Germany. [Es leben zu viele Ausländer in Deutschland.]
(3)	If jobs become scarce, the foreigners living in Germany should be sent back to their homeland. [Wenn Arbeitsplätze knapp werden, sollte man die in Deutschland lebenden Ausländer wieder in ihre Heimat zurückschicken]
(4)	(−) The Muslim culture can fit into our western world. [Die muslimische Kultur passt durchaus in unsere westliche Welt.]
(5)	(−) Islamic and Western European values can be reconciled. [Islamische und westeuropäische Wertvorstellungen lassen sich miteinander vereinbaren.]
(6)	(−) Islam fits perfectly into our western world. [Der Islam passt durchaus in unsere westliche Welt.]
(7)	Aussiedler (people from foreign countries of German origin) should be better off than foreigners because they are of German origin. [Aussiedler (deutschstämmige Ausländer) sollten bessergestellt werden als Ausländer, da sie deutscher Abstammung sind.]

(−) indicate reversed items. Original German wording in brackets.

We aggregated the responses of all seven items to one scale reflecting a positive attitude toward the group. The score was the mean of all seven responses for each participant. We aimed to reach a Cronbach’s α = 0.7 and preregistered that all items with a corrected item-total correlation <0.3 would be removed from the scale—in the end, none of the items reached this criterion, so we did not have to remove any data. The scale showed good consistency with Cronbach’s α = 0.89, *M* = 8.09 and *SD* = 1.71.

#### Social dominance orientation

To measure social dominance orientation, we used the German Version of the 4-item Short Social Dominance Orientation (SSDO; Pratto et al., 2013). This measure was not relevant to the current study.

#### Stimuli

**FIGURE 6:**
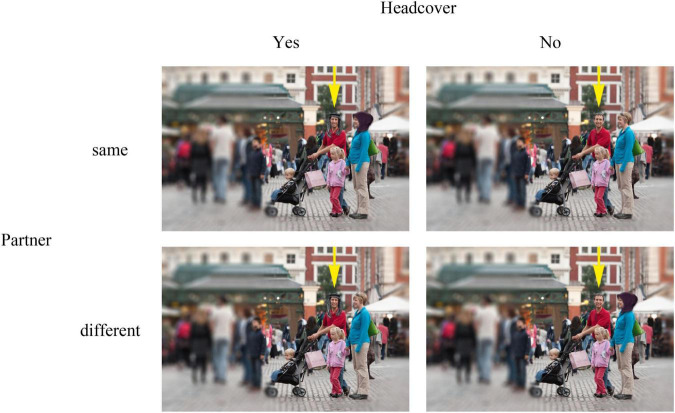
Stimuli used in experiment 2. The figure shows all variations for the male target person. For the female target person, the arrow was moved above the female. To ensure the anonymity of the people displayed in the photograph, faces were blurred for the published manuscript. The original photo (without head cover and blurring effects) was kindly made public by Jason Pier through CC BY-NC 2.0 license.

The photograph used as stimuli showed a small family consisting of two adults and two children. The stimuli were the same as in Experiment 1, the only difference being that the target persons wore hijabs (when female) and kufiyahs (when male) instead of masks (see Figure 6).

#### Dependent variables

The judgment on the character of the target person was measured with a 7-point rating scale (disagree – agree). The character is described in one sentence. The experiment was carried out in German, but the items here are presented in English (the original wording in German is given in parentheses in Table 4). All items were measured on a 7-point rating scale (1 = *fully disagree [trifft gar nicht zu]*, 7 = *fully agree [trifft vollkommen zu]*).

**TABLE 4:** Items measuring how the participant perceives the target person.

No	Item
(1)	The person thinks of herself first. [Die Person denkt zuerst an sich selbst.]
(2)	The person is calculating. [Die Person ist berechnend.]
(3)	The person is neurotic. [Die Person ist neurotisch.]
(4)	The person is aggressive. [Die Person ist aggressiv.]
(5)	The person is dangerous. [Die Person ist gefährlich.]
(6)	The person is a cynic. [Die Person ist zynisch]
(7)	The person is lazy. [Die Person ist faul.]
(8)	The person is arrogant. [Die Person ist arrogant.]
(9)	The person is very prosocial. [Die Person ist sehr prosozial.]
(10)	The person is balanced. [Die Person ist ausgeglichen.]
(11)	The person is concerned for others. [Die Person denkt an andere.]
(12)	The person is careful. [Die Person ist umsichtig.]
(13)	The person is trustworthy. [Die Person ist vertrauenswürdig.]
(14)	The person is industrious. [Die Person ist fleissig.]
(15)	The person is attractive. [Die Person ist attraktiv.]
(16)	The person is liked by me. [Die Person ist mir sympathisch.]

Here the English translation is shown. Original German wording in brackets.

Items 15 and 16 are DV for H1. Items 1–8 will be aggregated to a scale reflecting negative character traits, 9–14 positive character traits, and DV for H2. The reliability for the positive character traits was measured as Rcn = 0.52 and for negative character traits as Rcn = 0.38.

After responding to all pictures, we assessed the participants’ opinion about how representative a head cover is for the group by asking: The [picture of the clothing item] is a characteristic of people who consider themselves Muslims. [Das [Bild vom Kleidungsstück] ist ein Merkmal für Menschen, die dem Islam angehören]. The responses were measured on a rating scale of 1 (*do not agree*) – 10 (*totally agree*).

### Procedure

The procedure was the same as in Experiment 1.

### Statistical analysis

We used linear multilevel regressions with participants’ ID as a random effect to account for the repeated measures. For the analysis, we mean-centered the variable attitude toward the group and all factorial variables were dummy-coded. For multilevel linear regressions, we used the R package {lmer} (Bates et al., 2015). For analysis of slopes, we used {interactions} (Long, 2019). Because treating ordinal responses as continuous can result in wrong inferences (Liddell and Kruschke, 2018), we also report results based on an ordinal regression in a Bayesian framework in Electronic supplement F. In the present study, the results were robust.

#### Outliers and exclusions

As preregistered, we ran the analysis with the full data set. We carried out a second analysis in which we excluded participants who responded with a rating lower than 5 to the question: The—showing the picture of the clothing item—is a characteristic of people who consider themselves Muslims [Das—showing the picture of the clothing item—ist ein Merkmal für Menschen die dem Islam angehören]. The responses are measured on a rating scale of 1 (*do not agree*)—10 (*totally agree*). Because this would indicate that the participant does not associate a head cover with Muslim culture.

#### Deviations from the preregistration

We made a mistake in the analysis section. Our Hypothesis 2 is about positive and negative characteristics. In an earlier version, we had written aggression, prosociality, and self-interest. We also missed changing this in the analysis section.

We planned to carry out regressions for two distribution families: Gaussian and cumulative probit in a Bayesian framework and a frequentist framework. However, the ordinal model in a frequentist framework did not converge. For this reason, we decided to only fit the Bayesian models in the robustness check.

We assumed a homogeneous sample. However, inspecting the data shows that some participants show very extreme values in their responses on the scale measuring the attitude toward the group. Linear regression assumes a homogeneous sample and is biased by extreme values in the predictor variable, for this reason, we winsorized these values to –2 SDs for the centered variable.

## Results

### Test of preregistered hypothesis H1

**FIGURE 7:**
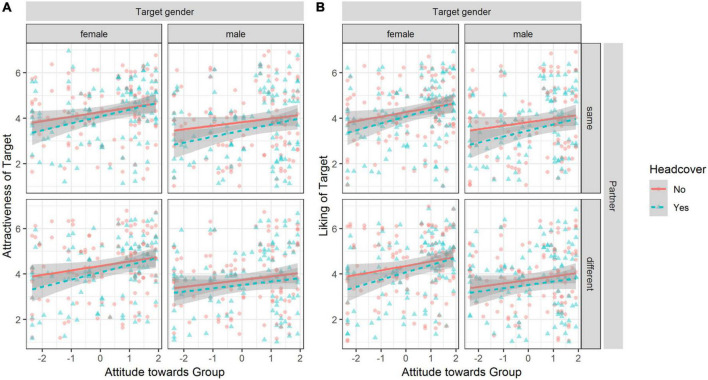
Observed attractiveness and liking ratings dependent on attitude, headcover, target person gender, and partner. Plot **(A)** for perceived attractiveness, plot **(B)** for liking. Lines depict linear regression of Y on X and the shaded areas show the 95% CI of the estimate. Points are jittered to avoid overlap.

We observed that perceived attractiveness and liking were dependent on the participants’ attitude toward measuring the group and whether the target person was wearing a head cover or not (see Figure 7). We fit regression models for attractiveness and liking individually (for all estimates, see Supplementary Table 7 in the Electronic supplement).

For attractiveness, a likelihood ratio test indicated that the interaction model accounting for wearing a head cover by the target person and attitude toward the group resulted in the best fit compared to the main effects only model, χ^2^ (1) = 3.91, *p* = 0.048, and fixed effects explained 8% of the variance (marg. *R*^2^ = 0.08). Adding the other interactions did not significantly increase fit. In the full factorial model, attractiveness was significantly positively affected by the attitude toward the group, β = 0.20, 95% CI [0.05, 0.35], and significantly decreased when the target person was male compared to a female target person, β = −0.19, 95% CI [−0.22, −0.15]. The moderation effect of the attitude toward the group was not significant, β = 0.11, 95% CI [−0.06, 0.27].

For liking, a likelihood ratio test indicated that the interaction model accounting for wearing a head cover by the target person and attitude toward the group resulted in the best fit compared to the main effects only model, χ^2^ (1) = 13.68, *p* < 0.001, and fixed effects explained 18% of the variance (marg. *R*^2^ = 0.18). Adding the other interactions did not significantly increase fit. In the full factorial model, liking was significantly positively affected by the attitude toward the group, β = 0.40, 95% CI [0.27, 0.52], and significantly decreased when the target person wore a headcover compared to no headcover, β = −0.11, 95% CI [−0.15, −0.07]. This effect was moderated by the attitude toward the group, β = 0.08, 95% CI [0.04, 0.12]. To test in which regions the slopes differ, we used the Johnson-Neyman procedure. When the moderator value, i.e., the attitude toward the group, was below the interval [0.72, 89.21], the liking of the target person was significantly lower when wearing a headcover than when wearing no headcover.

### Test of preregistered hypothesis H2

**FIGURE 8:**
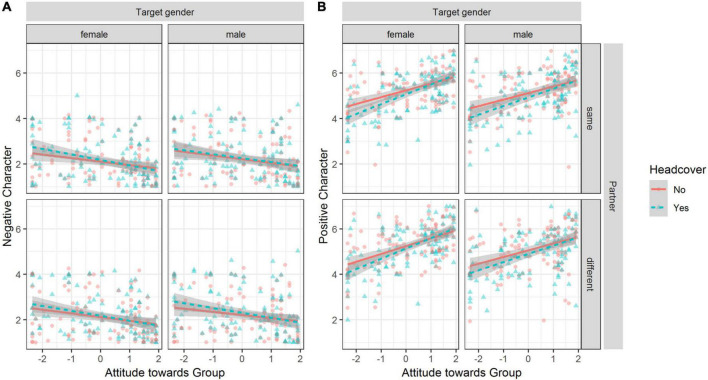
Observed positive and negative character judgments dependent on attitude, head cover, target person gender, and partner. Plot **(A)** for negative character judgments. Plot **(B)** for positive character judgments. Lines depict linear regression of Y on X; the shaded areas show the 95% CI of the estimate. Points are jittered to avoid overlap.

We observed that wearing a headcover influenced positive and negative character judgments dependent on the perceiver’s attitude toward the group (see Figure 8). We fit regression models for negative and positive character judgments individually (for all estimates, see Supplementary Table 8 in the Electronic supplement).

For negative character judgment, a likelihood ratio test indicated that the interaction model accounting for wearing a headcover by the target person and attitude toward the group resulted in the best fit compared to the main effects only model, χ^2^ (1) = 8.21, *p* = 0.004, and fixed effects explained 7% of the variance (marg. *R*^2^ = 0.07). Adding the other interactions did not significantly increase fit. The effect of wearing a headcover on negative character judgment, β = 0.03, 95% CI [−0.00, 0.06], was moderated by the attitude toward the group, β = −0.04, 95% CI [−0.07, −0.01]. To assess in which regions the slopes differ, we used the Johnson-Neyman procedure. When the moderator value of the attitude toward the group was below the interval [−0.55, 8.3], the judgments of the negative character of the target person were significantly higher when wearing a headcover than when wearing no headcover.

For positive character, a likelihood ratio test indicated that the interaction model accounting for wearing a headcover by the target person and attitude toward the group resulted in the best fit compared to the main effects only model, χ^2^ (1) = 19.64, *p* < 0.001, and fixed effects explained 26% of the variance (marg. *R*^2^ = 0.26). Adding the other interactions did not significantly increase fit. The effect of wearing a headcover on positive character judgment, β = −0.07, 95% CI [−0.10, −0.04], was moderated by the attitude toward the group, β = 0.07, 95% CI [0.04, 0.10]. When the moderator value of the attitude toward the group was below the interval [0.36, 3.96], the judgments of the positive character of the target person were significantly lower when wearing a headcover than when wearing no headcover.

### Robustness check

#### Ordinal Bayesian regression

To check the robustness of our results, we used a Bayesian analysis for ordinal linear regression. The detailed results can be found in Electronic supplement F. To test H1, we fit an ordinal multilevel model with data clustered in cases using a cumulative-probit distribution. For H1 regarding liking and attractiveness, the results show that the effects were of similar size and the same direction and an 83% HDI for liking [75% HDI for attractive] of the odds ratio of the interaction of wearing a headcover and attitude toward the group were smaller [greater] than 1. When attitude toward the group is negative, then a target person wearing a headcover receives lower ratings than when wearing no headcover.

To test H2, we fit an ordinal multilevel model with data clustered in cases and items using a cumulative-probit distribution. For H2 regarding negative and positive character, the results show that the effects were of similar size and the same direction as in the main analyses. A 95% HDI for the negative character [positive character] of the odds ratio of the interaction of wearing a headcover and attitude toward the group was smaller [greater] than 1. For negative character judgments, when the attitude toward the group is negative, a target person wearing a headcover receives higher ratings than when wearing no headcover; this effect decreases with increasing attitude toward the group. For a positive character judgment, when the attitude toward the group decreases, a target person wearing a headcover receives lower ratings than when wearing no headcover.

For all judgments, the effect of the difference between wearing a headcover or not decreases with increasing attitude toward the group.

#### Data exclusion

Based on the exclusion criteria, the perception of the head cover as paraphernalia of Muslim culture had to rate 5 for male and female headcovers, *n* = 32 participants had to be excluded. Re-running all our analyses showed that in the linear regressions, only the interaction of the attitude toward the group with wearing the headcover on the judgment of positive character did not include zero for the 95% CIs for the linear regression. For all other judgments, the effects were not robust in terms of statistical significance. However, the direction and size of the effects matched the full sample (see Electronic supplement G).

For the ordinal cumulative-probit regression, the size and directions of the effects were similar to the full sample, but the 95% Credible Intervals increased for attractiveness. The interaction effect postulated in H1 had a 78% HDI for liking and an 83% HDI for attractiveness for the odds ratio smaller than 1 for the interaction of wearing a headcover and attitude toward the group. For negative and positive character judgments, the effect postulated in H2 had 93% HDI of odds ratio being smaller than 1 for the negative and 95% HDI for odds ratio being larger than 1 for the positive character judgment.

Taken together, the exclusion of participants who did not perceive the headcovers as specific to the group did not alter the size or direction of effects, but it increased the uncertainty about the parameter estimates.

## Discussion

In two preregistered online studies, we examined the effect of facial coverings on person perception and judgment. In one study, we examined medical masks, and in a second study, we examined the effect of headcovers. We found that participants’ judgments of the person wearing the facial covering relied on the participants’ attitude toward issues associated with the facial covering, that is, its’ symbolic meaning. We revealed that this interaction effect occurs when wearing a face mask as well as wearing a headcover associated with Islam.

In both studies, we presented photographs of individuals in a day-to-day situation and asked participants to judge them along different dimensions (attractiveness, liking, and character). We varied the visual presentation of the individuals in the pictures by experimentally adding a facial mask typically associated with the context of a pandemic like COVID-19 (Experiment 1) or a headcover (stereo) typically associated with Muslim cultures (Experiment 2) to the faces. In Experiment 1, we measured participants’ attitudes toward COVID-19 (the COVID-19 pandemic was still relatively new, and many preventative measures were still active, therefore participants’ attitudes toward it were easily accessible to them as they ruled their daily lives), and in Experiment 2, we measured participants’ prejudice against Islam. In both studies, the judgments of individuals were altered depending on whether the target persons wore the respective facial covering and the participant’s attitude. Furthermore, we observed that the effect of masks on the judgment of the target person was stronger than the effect of wearing a head covering. This difference might be explained by the salience of the attitude associated with the facial covering. During data acquisition, the COVID-19 pandemic was on people’s minds resulting in high salience of attitudes that favor and oppose masks. On the other hand, the attitude toward individuals with Muslim backgrounds might have been less important to most people as this was not a focus topic of daily politics when the studies were conducted.

By showing that the same mechanism influences judgments of people wearing facial masks and people wearing head covers, we can generalize the effect of face covers on a person’s perception to more general processes underlying social cognition. Judgments are dependent on cues and the valuation of specific cues. And these cues are in the eye of the beholder, which is apparent in the large body of research showing the effect of stereotypes on person perception (Macrae and Bodenhausen, 2000).

Our hypotheses were derived from theories of social cognition and proposed that the attitude of the perceiver, together with the appearance of the human target person, determines the resulting judgments. Our studies resolve inconsistent results in the literature, e.g., that people wearing medical masks are judged more positively among students in some samples (Oldmeadow and Koch, 2021; Hies and Lewis, 2022) but more negatively in other student samples (Miyazaki and Kawahara, 2016). These seemingly contradicting results might be attributed to a further factor that was not considered before: the perceivers’ attitudes associated with wearing masks. Including a variable on the perceiver level allows us to frame the whole resulting pattern within a general theory of person perception. This highlights that perceptions and judgments of individuals rely on top-down information processing (Macrae and Bodenhausen, 2000), in the present experiments triggered by the associations held by the perceivers with certain head covers.

### Limitations and future research

Before generalizing these findings, important boundaries must be considered.

First, the judgment of the target persons did not matter to the participants. For this reason, participants might be more likely to use heuristic information processing (Pendry and Macrae, 1994), potentially rendering the personal attitude toward the facial covering more powerful. However, when people are facing strangers in everyday life, they also might not be motivated to process information carefully.

Second, the judgment task was highly artificial in that pictures were used and not a real interaction. The pictures were constructed by directly manipulating the appearance of the target persons. The judgment of the target persons was based on reported ratings and could have been influenced by social desirability.

Third, while experiments studying the effects on attractiveness usually utilize frontal pictures in the foreground, a landmark paper from 2007 showed that face and body attractiveness may convey different, and potentially independent, signals about an individual’s mate quality (Peters et al., 2007). In our study, we also have to take into account the esthetic properties of the clothes, and certainly, the esthetics of the head (or face) covers. From very recent face mask research, we do know that face masks modulate the attractiveness of faces as such, most generally, unattractive faces seem to benefit from covering the lower half of the face (Pazhoohi et al., 2021). Similar effects are to be expected with headcovers where covered parts of the head which are less attractive might boost the overall attractiveness of a face. Analogously, we could expect similar effects for covering less attractive body parts which might lead to the imagination of a whole body that is more attractive. Indications for this idea stem from research about veiled and non-veiled bodies and the degree of body-(dis)satisfaction (Wilhelm et al., 2018). Future research should explore how the different foci and perspectives used in the stimuli themselves influence person perception.

Fourth, as pointed out in the introduction, the perceived similarity is a strong predictor of liking and perceived attractiveness—while we aimed to manipulate attitude similarity, we did not measure perceived similarity, which would have functioned as a manipulation check. We decided not to use this measure to avoid demand effects; however, future research could replicate our experiments and additionally measure perceived similarity and test if our observed effects are explained through this process.

Finally, the participation was voluntary, and therefore the sample is not representative of the general population—which could have influenced the results. For example, individuals holding strong negative attitudes against COVID-19 or Muslims might not be willing to participate in research studies.

## Conclusion

Our results suggest that participants in our study did not judge the person depicted in the picture but judged the head covering and communicated their attitude toward its symbolic meaning. This suggests that the constructed representation of the person perceived could be more strongly influenced by cues and their associations, the stronger the attitude of the perceivers. This deeper consideration also explains why some people become aggressive with people wearing masks while others become relaxed and feel safe when attending to such persons—or react neutrally. Likewise, it explains why some people start to aggressively tear down others’ head covers while other observers’ imaginations of hospitality and warmth of Muslim cultures are triggered by the same symbol that is torn down by others. In the end, our assessments of others’ values, properties, and traits are deeply rooted in free associations emerging non-consciously which can hardly be controlled by the perceiver (see Ortlieb et al., 2020). To sum up, the present studies show that it is important to not only ask if and how certain face covers affect a person’s perception, but we should also ask what attitude the perceivers hold toward the facial covering’s symbolic meaning and linked associations.

## Data availability statement

The datasets presented in this study can be found in online repositories. The names of the repository/repositories and accession number(s) can be found below: https://osf.io/g8b75/.

## Ethics statement

Ethical review and approval was not required for the study on human participants in accordance with the local legislation and institutional requirements. The patients/participants provided their written informed consent to participate in this study.

## Author contributions

JL and C-CC conceived and implemented the experiments. JL analyzed and curated the data. JL and LK wrote the first draft of the manuscript. All authors revised the manuscript, contributed to the article, and approved the submitted version.

## Footnotes

1
https://www.antidiskriminierungsstelle.de/SharedDocs/downloads/DE/publikationen/Expertisen/expertise_diskr_aufgrund_islam_religionszugehoerigkeit_sozialwissenschaftlich.pdf?__blob=publicationFile&v=6
