# Supplementary material for: Perceptions of persons who wear face coverings are modulated by the perceivers’ attitude
Source: Front Neurosci. 2022 Nov 4;16:988546. doi: 10.3389/fnins.2022.988546 (PMC9672466; doi:10.3389/fnins.2022.988546)
Supplement: Supplementary file 1 [file Table_1.DOCX]

1. Supplements to
   Perceptions of persons who wear face coverings are modulated by the perceivers' attitude
   1. Table of Contents

[Supplement A – Experiment 1 Demographic Information of Participants 2](#_Toc110855070)

[Supplement B – Experiment 1 Linear Regression for H1 and H2 3](#_Toc110855071)

[Supplement C – Experiment 1 Ordinal Bayesian Regression 7](#_Toc110855072)

[Supplement D – Experiment 2 Demographic Information of Participants 11](#_Toc110855073)

[Supplement B – Experiment 2 Linear Regression for H1 and H2 12](#_Toc110855074)

[Supplement E – Experiment 2 Ordinal Bayesian Regression 19](#_Toc110855075)

[Supplement F – Experiment 2 Analyses After Applying Exclusion Criterion 23](#_Toc110855076)

Last edited: Thursday, August 11, 2022

- - 1. Supplement A – Experiment 1: Demographic Information of Participants

**Table S1**

*Demographic Description of Sample*

|  | Overall (*N* = 101) |
| --- | --- |
| Age |  |
| *N*-miss | 2 |
| Mean (SD) | 35.9 (19.0) |
| Range | 18 - 81 |
| Gender |  |
| *N*-miss | 2 |
| Female | 75 (75.8%) |
| Male | 23 (23.2%) |
| Diverse | 1 (1.0%) |
| Education |  |
| *N*-miss | 6 |
| No degree | 0 (0.0%) |
| Still in school | 0 (0.0%) |
| Primary School | 0 (0.0%) |
| Secondary School | 2 (2.1%) |
| Apprenticeship | 5 (5.3%) |
| Advanced technical college qualification | 5 (5.3%) |
| Abitur | 61 (64.2%) |
| University Degree | 22 (23.2%) |
| other | 0 (0.0%) |
| No response | 0 (0.0%) |

Supplement B – Experiment 1: Linear Regression for H1 and H2

**Table S2**

*Results of the Regression of Attractiveness and Liking on Experimental Factors Testing H1*

|  | Attractiveness | | | | Liking | | | |  |
| --- | --- | --- | --- | --- | --- | --- | --- | --- | --- |
| Predictors | *B* | *B* 95% CI | β | β 95% CI | *B* | *B* 95% CI | β | β 95% CI | |
| Int. | 3.52 ** | 3.26; 3.78 | -0.00 | -0.15; 0.15 | 4.19 ** | 3.91; 4.47 | 0.00 | -0.13; 0.13 | |
| Attitude COVID-19 (1) | -0.18 | -0.51; 0.15 | -0.03 | -0.18; 0.12 | -0.77 ** | -1.12; -0.41 | -0.06 | -0.18; 0.07 | |
| Mask (2) | -0.10 | -0.35; 0.15 | -0.00 | -0.05; 0.04 | 0.25 | -0.06; 0.56 | 0.14 | 0.09; 0.20 | |
| Male (3) | -0.23 | -0.48; 0.02 | -0.09 | -0.14; -0.04 | -0.24 | -0.55; 0.07 | -0.05 | -0.10; -0.00 | |
| Partner different (4) | -0.04 | -0.29; 0.21 | -0.01 | -0.06; 0.03 | -0.31 * | -0.62; -0.00 | -0.06 | -0.11; -0.01 | |
| 1 x 2 | 0.42 * | 0.09; 0.74 | 0.12 | 0.07; 0.17 | 1.35 ** | 0.95; 1.74 | 0.31 | 0.26; 0.36 | |
| 1 x 3 | -0.13 | -0.45; 0.19 | -0.06 | -0.10; -0.01 | -0.04 | -0.43; 0.35 | -0.02 | -0.07; 0.04 | |
| 2 x 3 | 0.07 | -0.29; 0.43 | 0.02 | -0.03; 0.06 | 0.15 | -0.29; 0.59 | 0.02 | -0.03; 0.07 | |
| 1 x 4 | -0.05 | -0.37; 0.27 | -0.00 | -0.05; 0.05 | 0.12 | -0.28; 0.51 | 0.01 | -0.04; 0.06 | |
| 2 x 4 | 0.09 | -0.27; 0.45 | 0.02 | -0.03; 0.07 | 0.25 | -0.19; 0.69 | 0.04 | -0.01; 0.09 | |
| 3 x 4 | -0.10 | -0.46; 0.26 | -0.02 | -0.06; 0.03 | 0.05 | -0.39; 0.49 | 0.00 | -0.05; 0.05 | |
| 1 x 2 x 3 | -0.08 | -0.53; 0.38 | -0.03 | -0.07; 0.02 | -0.08 | -0.63; 0.48 | -0.02 | -0.07; 0.03 | |
| 1 x 2 x 4 | 0.13 | -0.33; 0.58 | 0.01 | -0.04; 0.05 | -0.18 | -0.74; 0.38 | -0.03 | -0.09; 0.02 | |
| 1 x 3 x 4 | 0.05 | -0.40; 0.51 | -0.01 | -0.05; 0.04 | 0.12 | -0.44; 0.67 | 0.00 | -0.05; 0.06 | |
| 2 x 3 x 4 | 0.03 | -0.47; 0.53 | 0.00 | -0.04; 0.05 | -0.06 | -0.68; 0.56 | -0.00 | -0.06; 0.05 | |
| 1 x 2 x 3 x 4 | -0.18 | -0.82; 0.46 | -0.01 | -0.06; 0.03 | -0.16 | -0.95; 0.62 | -0.01 | -0.06; 0.04 | |
| Random Effects | | | | | | | | |  |
| σ2 | 0.83 | | | | 1.25 | | | |  |
| τ00 | 0.91 | | | | 0.79 | | | |  |
| ICC | 0.52 | | | | 0.39 | | | |  |
| *N* | 100 | | | | 100 | | | |  |
| Observations | 800 | | | | 800 | | | |  |
| Marginal *R*^2^ / Conditional *R*^2^ | 0.028 / 0.538 | | | | 0.126 / 0.464 | | | |  |
| AIC | 2,404.631 | | | | 2,679.859 | | | |  |
| log-Likelihood | -1,184.315 | | | | -1,321.930 | | | |  |
| *Note. * p<0.05   ** p<0.01* | | | | | | | | |  |

**Table S3**

*Results of the Regression of Attractiveness and Liking on Experimental Factors Testing H2*

|  | **Conformity** | | | | **Prosociality** | | | | **Self-interest** | | | |  |
| --- | --- | --- | --- | --- | --- | --- | --- | --- | --- | --- | --- | --- | --- |
| *Predictors* | *B* | *B* 95% CI | β | β 95% CI | *B* | *B* 95% CI | β | β 95% CI | *B* | *B* 95% CI | β | β 95% CI |  |
| Int. | 3.00 ^**^ | 2.76; 3.24 | -0.00 | -0.09; 0.09 | 2.47 ^**^ | 2.26; 2.67 | -0.00 | -0.07; 0.07 | 1.88 ^**^ | 1.67; 2.10 | 0.00 | -0.09; 0.09 |  |
| Attitude COVID-19 (1) | -0.17 | -0.47; 0.14 | -0.05 | -0.14; 0.04 | -0.55 ^**^ | -0.81; -0.29 | 0.00 | -0.07; 0.07 | -0.31 ^*^ | -0.58; -0.03 | -0.10 | -0.19; -0.01 |  |
| Mask (2) | 1.91 ^**^ | 1.61; 2.20 | 0.62 | 0.58; 0.67 | 2.33 ^**^ | 2.07; 2.58 | 0.75 | 0.71; 0.79 | 2.07 ^**^ | 1.82; 2.33 | 0.67 | 0.63; 0.72 |  |
| Male (3) | -0.08 | -0.37; 0.22 | 0.01 | -0.04; 0.06 | -0.09 | -0.34; 0.17 | -0.01 | -0.05; 0.03 | -0.05 | -0.30; 0.21 | -0.02 | -0.06; 0.02 |  |
| Partner different (4) | -0.29 | -0.59; 0.00 | -0.05 | -0.09; 0.00 | -0.16 | -0.42; 0.09 | -0.02 | -0.06; 0.02 | 0.04 | -0.22; 0.30 | 0.04 | -0.00; 0.08 |  |
| 1 x 2 | 0.13 | -0.24; 0.51 | -0.04 | -0.09; 0.01 | 1.15 ^**^ | 0.83; 1.47 | 0.22 | 0.18; 0.26 | 0.30 | -0.02; 0.63 | 0.06 | 0.01; 0.10 |  |
| 1 x 3 | 0.21 | -0.17; 0.59 | 0.00 | -0.05; 0.05 | 0.13 | -0.20; 0.45 | -0.00 | -0.04; 0.04 | 0.08 | -0.25; 0.40 | -0.02 | -0.06; 0.02 |  |
| 2 x 3 | 0.12 | -0.30; 0.54 | -0.02 | -0.07; 0.03 | 0.11 | -0.25; 0.47 | 0.01 | -0.03; 0.04 | -0.13 | -0.49; 0.23 | -0.02 | -0.06; 0.02 |  |
| 1 x 4 | 0.25 | -0.13; 0.63 | -0.00 | -0.05; 0.04 | 0.18 | -0.14; 0.50 | -0.01 | -0.05; 0.03 | 0.02 | -0.31; 0.35 | -0.01 | -0.06; 0.03 |  |
| 2 x 4 | 0.22 | -0.20; 0.64 | -0.00 | -0.05; 0.04 | 0.22 | -0.14; 0.58 | 0.02 | -0.02; 0.06 | 0.04 | -0.32; 0.40 | 0.01 | -0.04; 0.05 |  |
| 3 x 4 | 0.32 | -0.10; 0.74 | 0.01 | -0.03; 0.06 | 0.05 | -0.31; 0.41 | -0.00 | -0.04; 0.04 | 0.11 | -0.25; 0.47 | 0.02 | -0.02; 0.06 |  |
| 1 x 2 x 3 | -0.40 | -0.93; 0.14 | -0.01 | -0.06; 0.04 | -0.21 | -0.66; 0.25 | -0.01 | -0.05; 0.03 | -0.18 | -0.64; 0.28 | -0.02 | -0.06; 0.02 |  |
| 1 x 2 x 4 | -0.50 | -1.03; 0.04 | -0.02 | -0.07; 0.03 | -0.40 | -0.85; 0.06 | -0.03 | -0.07; 0.01 | -0.01 | -0.47; 0.45 | 0.00 | -0.04; 0.04 |  |
| 1 x 3 x 4 | -0.35 | -0.89; 0.18 | -0.00 | -0.05; 0.04 | -0.20 | -0.65; 0.26 | -0.01 | -0.05; 0.03 | -0.15 | -0.61; 0.31 | -0.02 | -0.06; 0.02 |  |
| 2 x 3 x 4 | -0.48 | -1.08; 0.12 | -0.04 | -0.09; 0.01 | -0.14 | -0.64; 0.37 | -0.01 | -0.05; 0.03 | -0.00 | -0.52; 0.51 | -0.00 | -0.04; 0.04 |  |
| 1 x 2 x 3 x 4 | 0.65 | -0.10; 1.41 | 0.04 | -0.01; 0.09 | 0.26 | -0.38; 0.90 | 0.02 | -0.02; 0.05 | 0.03 | -0.63; 0.68 | 0.00 | -0.04; 0.04 |  |
| **Random Effects** | | | | | | | | | | | | | |
| σ^2^ | 1.15 | | | | 0.83 | | | | 0.85 | | | |  |
| τ_00_ | 0.34 | | | | 0.25 | | | | 0.38 | | | |  |
| ICC | 0.23 | | | | 0.23 | | | | 0.31 | | | |  |
| *N* | 100 | | | | 100 | | | | 100 | | | |  |
| Observations | 800 | | | | 800 | | | | 800 | | | |  |
| Marginal *R*^2^ / Conditional *R*^2^ | 0.397 / 0.536 | | | | 0.604 / 0.695 | | | | 0.465 / 0.629 | | | |  |
| AIC | 2,560.305 | | | | 2,306.527 | | | | 2,353.160 | | | |  |
| log-Likelihood | -1,262.152 | | | | -1,135.263 | | | | -1,158.580 | | | |  |
| *Note. * p<0.05   ** p<0.01* | | | | | | | | | | | | | |

- - 1. Supplement C – Experiment 1: Ordinal Bayesian Regression

**Table S4**

*Results of the Ordinal Regression of Attractiveness and Liking on Experimental Factors Testing H1*

|  | **Attractiveness** | | | **Liking** | | |
| --- | --- | --- | --- | --- | --- | --- |
| *Predictors* | *Odds Ratios* | *std. Error* | *CI (95%)* | *Odds Ratios* | *std. Error* | *CI (95%)* |
| Attitude Covid-19 (1) | 0.78 | 0.17 | 0.52,1.17 | 0.46 | 0.08 | 0.33,0.66 |
| Mask (2) | 0.89 | 0.14 | 0.67,1.21 | 1.28 | 0.19 | 0.95,1.73 |
| Male (3) | 0.75 | 0.12 | 0.55,1.02 | 0.79 | 0.12 | 0.59,1.06 |
| Partner different (4) | 0.96 | 0.15 | 0.71,1.30 | 0.75 | 0.11 | 0.56,1.01 |
| 1 x 2 | 1.67 | 0.32 | 1.15,2.45 | 3.64 | 0.71 | 2.51,5.27 |
| 1 x 3 | 0.86 | 0.17 | 0.58,1.26 | 0.96 | 0.18 | 0.66,1.41 |
| 2 x 3 | 1.08 | 0.24 | 0.70,1.66 | 1.14 | 0.25 | 0.75,1.74 |
| 1 x 4 | 0.97 | 0.19 | 0.66,1.44 | 1.14 | 0.22 | 0.79,1.67 |
| 2 x 4 | 1.10 | 0.23 | 0.72,1.69 | 1.25 | 0.26 | 0.82,1.91 |
| 3 x 4 | 0.88 | 0.19 | 0.57,1.36 | 1.07 | 0.23 | 0.70,1.62 |
| 1 x 2 x 3 | 0.92 | 0.24 | 0.53,1.54 | 0.94 | 0.26 | 0.55,1.61 |
| 1 x 2 x 4 | 1.14 | 0.31 | 0.67,1.99 | 0.84 | 0.22 | 0.49,1.42 |
| 1 x 3 x 4 | 1.05 | 0.28 | 0.61,1.77 | 1.11 | 0.31 | 0.65,1.90 |
| 2 x 3 x 4 | 1.06 | 0.32 | 0.57,1.94 | 0.95 | 0.29 | 0.53,1.80 |
| 1 x 2 x 3 x 4 | 0.79 | 0.29 | 0.36,1.66 | 0.85 | 0.32 | 0.40,1.82 |
| **Random Effects** | | | | | | |
| σ^2^ | 0.78 | | | 0.69 | | |
| τ_00_ | 0.99 | | | 1.61 | | |
| ICC | 0.44 | | | 0.30 | | |
| *N* | 100 _CASE_ | | | 100 _CASE_ | | |
| Observations | 800 | | | 800 | | |

*Note.* The regression was fit using non-informative uniform prior assuming a cumulative ordered probit distribution. When calculating R² predictions are treated as continuous variables which is likely invalid for ordinal families for this reason we do not show R².

**Figure S1**

*Marginal Effects Plot for the Interaction between Attitude Towards Covid-19 Measures and Mask for Attractiveness*


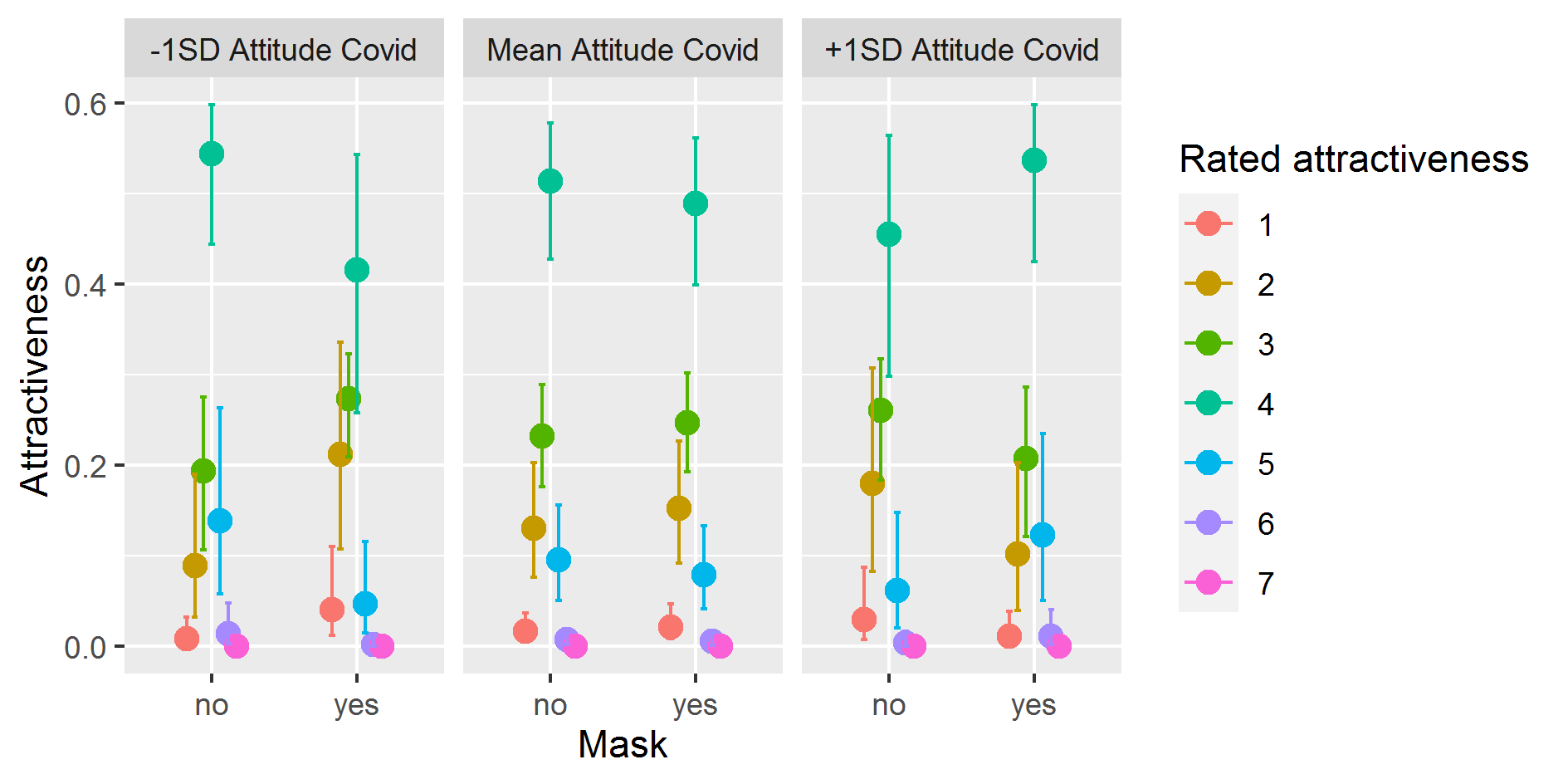


**Figure S2**

*Marginal Effects Plot for the Interaction between Attitude Towards Covid-19 Measures and Mask for Liking*


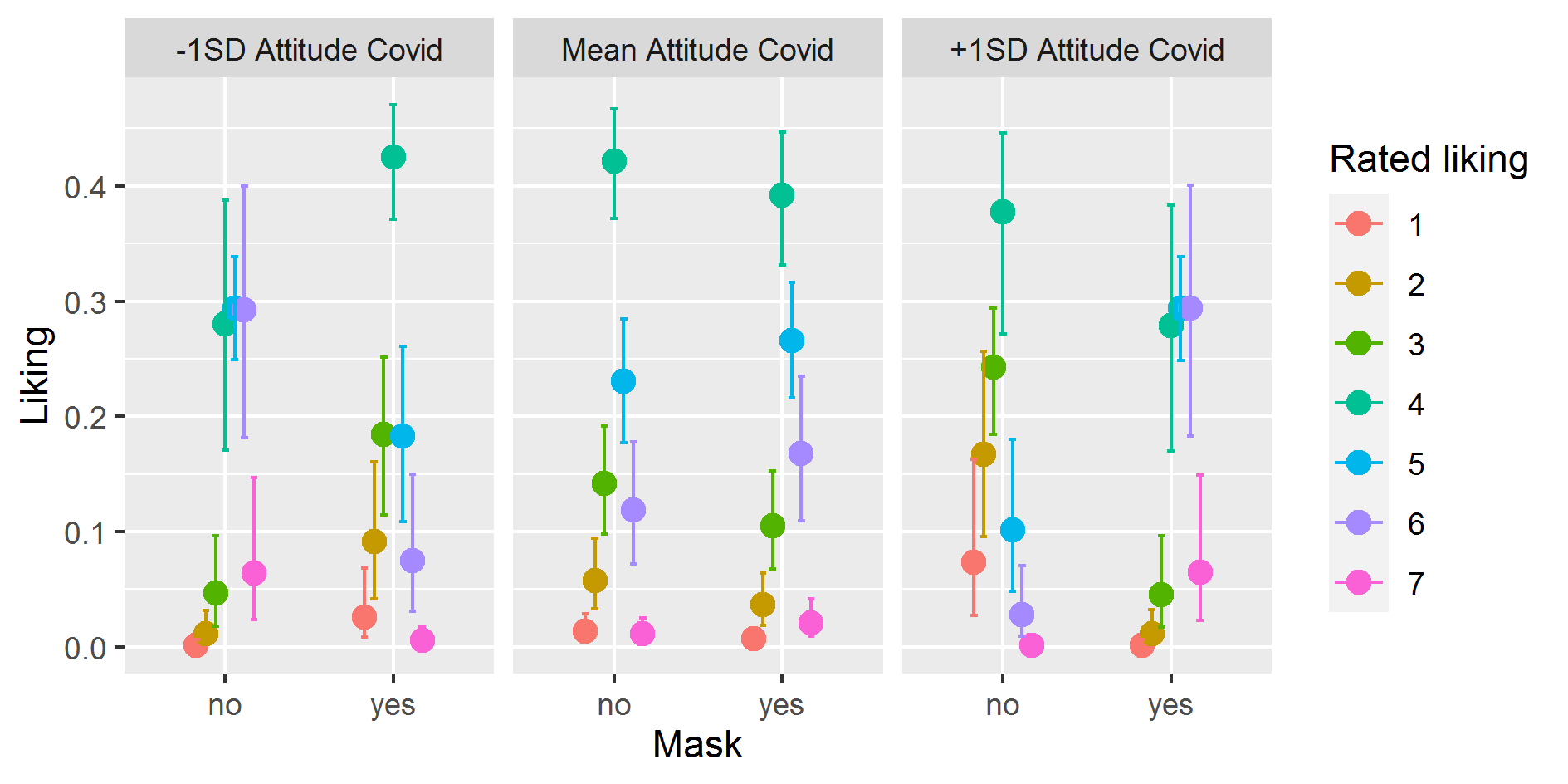


**Table S5**

*Results of the Ordinal Regression of Conformity, Prosociality and Selfishness on Experimental Factors Testing H2*

|  | **Conformity** | | | **Prosociality** | | | **Self-interest** | | |
| --- | --- | --- | --- | --- | --- | --- | --- | --- | --- |
| *Predictors* | *Odds Ratios* | *std. Error* | *CI (95%)* | *Odds Ratios* | *std. Error* | *CI (95%)* | *Odds Ratios* | *std. Error* | *CI (95%)* |
| Attitude Covid-19 (1) | 0.90 | 0.08 | 0.76,1.09 | 0.62 | 0.06 | 0.52,0.75 | 0.73 | 0.08 | 0.59,0.91 |
| Mask (2) | 3.24 | 0.28 | 2.73,3.81 | 5.51 | 0.52 | 4.59,6.63 | 4.66 | 0.43 | 3.86,5.62 |
| Male (3) | 0.97 | 0.08 | 0.81,1.15 | 0.94 | 0.09 | 0.79,1.12 | 0.97 | 0.10 | 0.80,1.17 |
| Partner different (4) | 0.85 | 0.07 | 0.72,1.02 | 0.91 | 0.09 | 0.76,1.11 | 1.06 | 0.10 | 0.87,1.29 |
| 1 x 2 | 1.11 | 0.13 | 0.91,1.38 | 2.53 | 0.29 | 2.04,3.21 | 1.40 | 0.16 | 1.10,1.75 |
| 1 x 3 | 1.14 | 0.13 | 0.92,1.42 | 1.10 | 0.13 | 0.88,1.39 | 1.06 | 0.13 | 0.83,1.32 |
| 2 x 3 | 1.07 | 0.13 | 0.85,1.38 | 1.10 | 0.14 | 0.87,1.41 | 0.93 | 0.12 | 0.73,1.21 |
| 1 x 4 | 1.18 | 0.13 | 0.95,1.47 | 1.17 | 0.13 | 0.93,1.46 | 1.02 | 0.13 | 0.80,1.31 |
| 2 x 4 | 1.15 | 0.14 | 0.91,1.46 | 1.15 | 0.15 | 0.90,1.47 | 1.01 | 0.14 | 0.77,1.31 |
| 3 x 4 | 1.20 | 0.15 | 0.94,1.54 | 1.00 | 0.13 | 0.78,1.29 | 1.08 | 0.15 | 0.83,1.41 |
| 1 x 2 x 3 | 0.78 | 0.12 | 0.57,1.04 | 0.89 | 0.14 | 0.64,1.21 | 0.88 | 0.14 | 0.65,1.20 |
| 1 x 2 x 4 | 0.71 | 0.11 | 0.52,0.96 | 0.75 | 0.12 | 0.55,1.02 | 0.96 | 0.16 | 0.71,1.32 |
| 1 x 3 x 4 | 0.80 | 0.13 | 0.58,1.09 | 0.83 | 0.14 | 0.60,1.16 | 0.88 | 0.15 | 0.63,1.24 |
| 2 x 3 x 4 | 0.73 | 0.13 | 0.52,1.04 | 0.91 | 0.16 | 0.64,1.28 | 0.97 | 0.18 | 0.68,1.37 |
| 1 x 2 x 3 x 4 | 1.55 | 0.35 | 0.99,2.42 | 1.21 | 0.28 | 0.78,1.89 | 1.07 | 0.25 | 0.69,1.68 |
| **Random Effects** | | | | | | | | | |
| σ^2^ | 0.30 | | | 0.42 | | | 0.24 | | |
| τ_00_ | 3.78 | | | 3.87 | | | 3.57 | | |
| ICC | 0.07 | | | 0.10 | | | 0.06 | | |
| *N* | 100 _CASE_ | | | 100 _CASE_ | | | 100 _CASE_ | | |
|  | 3 _question_ | | | 3 _question_ | | | 3 _question_ | | |
| Observations | 2400 | | | 2400 | | | 2400 | | |

*Note.* The regression was fit using non-informative uniform prior assuming a cumulative ordered probit distribution. When calculating R² predictions are treated as continuous variables which is likely invalid for ordinal families for this reason we do not show R².

**Figure S3**

*Marginal Effects Plot for the Interaction between Attitude Towards Covid-19 Measures and Mask for Prosociality*

**
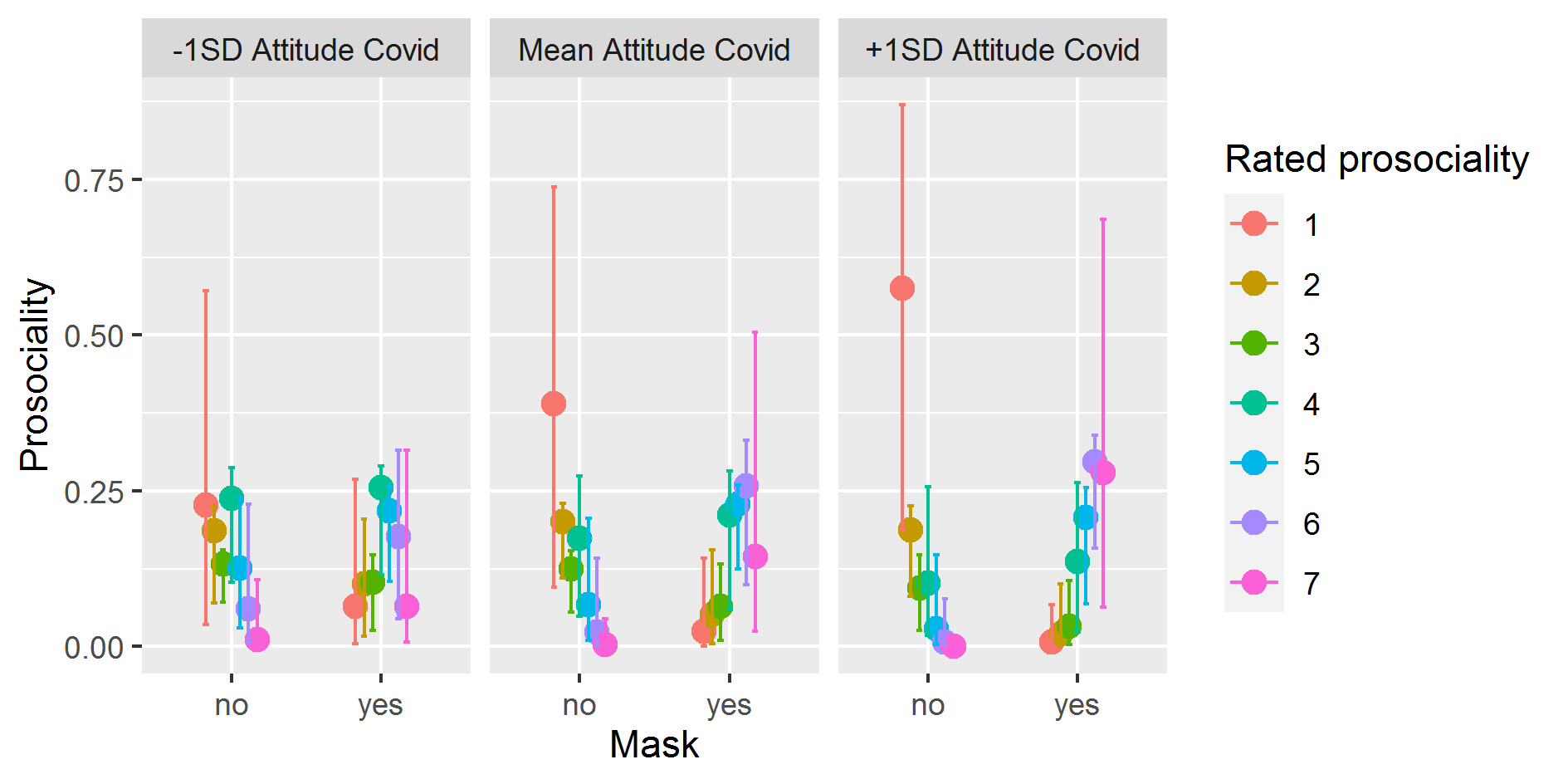
**

**Figure S4**

*Marginal Effects Plot for the Interaction between Attitude Towards Covid-19 Measures and Mask for Self-interest*

**
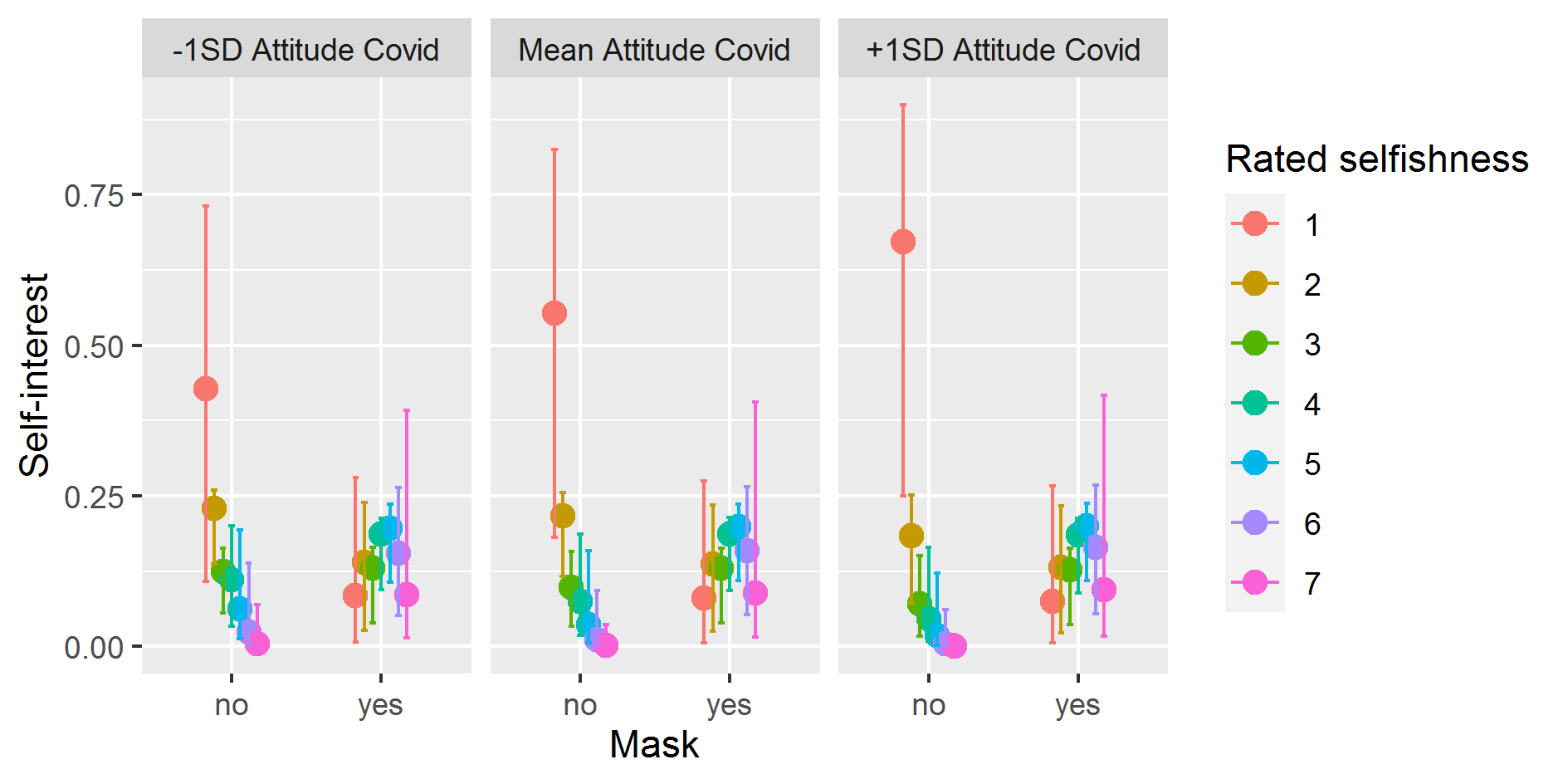
**

- - 1. Supplement D – Experiment 2: Demographic Information of Participants

**Table S6**

*Demographic Description of Sample*

| Overall (*N*=114) | |
| --- | --- |
| Age |  |
| N-Miss | 1 |
| Mean (SD) | 35.027 (17.241) |
| Range | 18.000 - 71.000 |
| Gender |  |
| Female | 89 (78.1%) |
| Male | 24 (21.1%) |
| Diverse | 1 (0.9%) |
| Education |  |
| N-Miss | 1 |
| No degree | 0 (0.0%) |
| Still in school | 0 (0.0%) |
| Primary School | 1 (0.9%) |
| Secondary School | 3 (2.7%) |
| Apprenticeship | 10 (8.8%) |
| Adv. technical college qualification | 5 (4.4%) |
| Abitur | 69 (61.1%) |
| University Degree | 25 (22.1%) |
| other | 0 (0.0%) |
| No response | 0 (0.0%) |

- - 1. Supplement E – Experiment 2: Linear Regression for H1 and H2

**Table S7**

Regression Results for the Full Factorial Models

|  | **Attractiveness** | | | | **Liking** | | | |
| --- | --- | --- | --- | --- | --- | --- | --- | --- |
| *Predictors* | *B* | *B* 95% CI | β | β 95% CI | *B* | *B* CI 95% | β | β 95% CI |
| Int. | 4.27 ^**^ | 4.00;4.54 | -0.00 | -0.15;0.15 | 5.36 ^**^ | 5.15;5.58 | -0.00 | -0.13;0.13 |
| Attitude Group (1) | 0.20 ^*^ | 0.00;0.39 | 0.20 | 0.05;0.35 | 0.29 ^**^ | 0.13;0.44 | 0.40 | 0.27;0.52 |
| Headcover (2) | -0.19 | -0.42;0.04 | -0.08 | -0.12;-0.05 | -0.34 ^**^ | -0.55;-0.13 | -0.11 | -0.15;-0.07 |
| Male (3) | -0.44 ^**^ | -0.67;-0.21 | -0.19 | -0.22;-0.15 | -0.14 | -0.35;0.07 | -0.08 | -0.12;-0.04 |
| Partner different (4) | 0.09 | -0.14;0.32 | 0.00 | -0.03;0.04 | 0.00 | -0.21;0.21 | -0.00 | -0.04;0.04 |
| 1 x 2 | 0.11 | -0.06;0.27 | 0.04 | 0.00;0.07 | 0.15 ^*^ | 0.00;0.30 | 0.08 | 0.04;0.12 |
| 1 x 3 | -0.04 | -0.21;0.12 | -0.03 | -0.07;0.00 | -0.06 | -0.21;0.09 | -0.05 | -0.09;-0.01 |
| 2 x 3 | -0.18 | -0.50;0.15 | -0.01 | -0.05;0.03 | -0.04 | -0.33;0.26 | 0.01 | -0.03;0.05 |
| 1 x 4 | 0.00 | -0.16;0.16 | -0.01 | -0.05;0.03 | 0.10 | -0.05;0.25 | 0.03 | -0.01;0.07 |
| 2 x 4 | -0.09 | -0.41;0.23 | 0.00 | -0.03;0.04 | 0.04 | -0.25;0.34 | 0.02 | -0.02;0.06 |
| 3 x 4 | -0.17 | -0.50;0.15 | -0.01 | -0.05;0.03 | -0.15 | -0.45;0.14 | -0.02 | -0.06;0.02 |
| 1 x 2 x 3 | 0.00 | -0.23;0.24 | -0.01 | -0.05;0.02 | 0.01 | -0.20;0.22 | -0.01 | -0.05;0.03 |
| 1 x 2 x 4 | 0.02 | -0.21;0.25 | -0.01 | -0.05;0.03 | 0.01 | -0.20;0.23 | -0.01 | -0.05;0.03 |
| 1 x 3 x 4 | -0.00 | -0.24;0.23 | -0.02 | -0.05;0.02 | -0.04 | -0.26;0.17 | -0.02 | -0.06;0.02 |
| 2 x 3 x 4 | 0.24 | -0.22;0.69 | 0.02 | -0.02;0.06 | 0.14 | -0.27;0.56 | 0.01 | -0.03;0.05 |
| 1 x 2 x 3 x 4 | -0.13 | -0.46;0.20 | -0.02 | -0.05;0.02 | -0.09 | -0.39;0.21 | -0.01 | -0.05;0.03 |
| **Random Effects** | | | | | | | | |
| σ^2^ | 0.77 | | | | 0.64 | | | |
| τ_00_ | 1.39 | | | | 0.74 | | | |
| ICC | 0.64 | | | | 0.53 | | | |
| *N* | 114 | | | | 114 | | | |
| Observations | 912 | | | | 912 | | | |
| Marginal *R*^2^ / Conditional *R*^2^ | 0.084 / 0.674 | | | | 0.184 / 0.620 | | | |
| AIC | 2,727.842 | | | | 2,522.615 | | | |
| log-Likelihood | -1,345.921 | | | | -1,243.308 | | | |
| *Note.* B shows the unstandardized regression coefficients. β shows the standardized regression coefficients.** p<0.05   ** p<0.01.* | | | | | | | | |

**Table S8**

*Regression Results for the Full Factorial Models*

|  | **Negative Character** | | | | **Positive Character** | | | |
| --- | --- | --- | --- | --- | --- | --- | --- | --- |
| *Predictors* | *B* | *B 9*5% CI | β | β 95% CI | *B* | *B* 95% CI | β | β 95% CI |
| Int. | 2.11 ^**^ | 1.93; 2.28 | 0.00 | -0.16; 0.16 | 5.25 ^**^ | 5.09; 5.40 | -0.00 | -0.14; 0.14 |
| Attitude Group (1) | -0.16 ^*^ | -0.28; -0.03 | -0.26 | -0.42; -0.10 | 0.30 ^**^ | 0.19; 0.41 | 0.49 | 0.36; 0.63 |
| Headcover (2) | 0.07 | -0.04; 0.18 | 0.03 | 0.00; 0.06 | -0.17 ^**^ | -0.30; -0.05 | -0.07 | -0.10; -0.04 |
| Male (3) | 0.10 | -0.01; 0.21 | 0.05 | 0.02; 0.08 | -0.12 | -0.24; 0.00 | -0.09 | -0.12; -0.06 |
| Partner different (4) | 0.02 | -0.09; 0.12 | 0.01 | -0.02; 0.04 | -0.00 | -0.12; 0.12 | -0.00 | -0.03; 0.03 |
| 1 x 2 | -0.09 ^*^ | -0.17; -0.01 | -0.04 | -0.07; -0.01 | 0.13 ^**^ | 0.05; 0.22 | 0.07 | 0.04; 0.10 |
| 1 x 3 | -0.01 | -0.09; 0.07 | 0.02 | -0.01; 0.04 | -0.01 | -0.10; 0.08 | -0.04 | -0.07; -0.00 |
| 2 x 3 | -0.03 | -0.18; 0.13 | 0.00 | -0.03; 0.03 | -0.02 | -0.19; 0.16 | -0.01 | -0.04; 0.02 |
| 1 x 4 | -0.01 | -0.09; 0.07 | 0.00 | -0.03; 0.03 | 0.04 | -0.04; 0.13 | 0.01 | -0.02; 0.04 |
| 2 x 4 | -0.02 | -0.17; 0.14 | 0.00 | -0.02; 0.03 | 0.07 | -0.10; 0.24 | 0.01 | -0.02; 0.04 |
| 3 x 4 | -0.01 | -0.17; 0.14 | 0.00 | -0.02; 0.03 | -0.06 | -0.24; 0.11 | -0.02 | -0.05; 0.01 |
| 1 x 2 x 3 | 0.08 | -0.04; 0.19 | 0.01 | -0.02; 0.04 | -0.04 | -0.17; 0.08 | -0.01 | -0.05; 0.02 |
| 1 x 2 x 4 | 0.03 | -0.08; 0.14 | -0.01 | -0.03; 0.02 | -0.03 | -0.15; 0.10 | -0.01 | -0.04; 0.02 |
| 1 x 3 x 4 | 0.03 | -0.08; 0.15 | -0.01 | -0.03; 0.02 | -0.04 | -0.16; 0.09 | -0.01 | -0.04; 0.02 |
| 2 x 3 x 4 | 0.06 | -0.16; 0.28 | 0.01 | -0.02; 0.03 | -0.04 | -0.28; 0.21 | -0.00 | -0.04; 0.03 |
| 1 x 2 x 3 x 4 | -0.10 | -0.26; 0.06 | -0.02 | -0.05; 0.01 | -0.00 | -0.18; 0.18 | -0.00 | -0.03; 0.03 |
| **Random Effects** | | | | | | | | |
| σ^2^ | 0.18 | | | | 0.22 | | | |
| τ_00_ | 0.72 | | | | 0.52 | | | |
| ICC | 0.80 | | | | 0.70 | | | |
| *N* | 114 | | | | 114 | | | |
| Observations | 912 | | | | 912 | | | |
| Marginal *R*^2^ / Conditional *R*^2^ | 0.072 / 0.818 | | | | 0.262 / 0.780 | | | |
| AIC | 1,499.047 | | | | 1,646.443 | | | |
| log-Likelihood | -731.523 | | | | -805.222 | | | |
| *Note.* B shows the unstandardized regression coefficients. β shows the standardized regression coefficients.** p*<0.05,,*** p<*0.01*.* | | | | | | | | |

Supplement F – Experiment 2: Ordinal Bayesian Regression

**Table S9**

*Results of the Ordinal Regression of Attractiveness and Liking on Experimental Factors Testing H1*

|  | **Attractiveness** | | | **Liking** | | |
| --- | --- | --- | --- | --- | --- | --- |
| *Predictors* | *Odds Ratios* | *std. Error* | *CI (95%)* | *Odds Ratios* | *std. Error* | *CI (95%)* |
| Attitude group (1) | 1.34 | 0.17 | 1.05,1.76 | 1.58 | 0.19 | 1.25,2.01 |
| Head cover (2) | 0.80 | 0.12 | 0.60,1.07 | 0.64 | 0.09 | 0.47,0.85 |
| Male (3) | 0.60 | 0.09 | 0.45,0.80 | 0.80 | 0.12 | 0.61,1.07 |
| Partner different (4) | 1.15 | 0.17 | 0.87,1.55 | 1.10 | 0.16 | 0.82,1.48 |
| 1 x 2 | 1.14 | 0.12 | 0.94,1.41 | 1.17 | 0.13 | 0.94,1.44 |
| 1 x 3 | 0.97 | 0.10 | 0.78,1.19 | 0.91 | 0.10 | 0.73,1.12 |
| 2 x 3 | 0.76 | 0.16 | 0.50,1.14 | 0.92 | 0.19 | 0.61,1.39 |
| 1 x 4 | 0.99 | 0.10 | 0.81,1.23 | 1.16 | 0.12 | 0.93,1.43 |
| 2 x 4 | 0.86 | 0.18 | 0.57,1.31 | 1.02 | 0.21 | 0.69,1.52 |
| 3 x 4 | 0.75 | 0.16 | 0.50,1.12 | 0.73 | 0.15 | 0.49,1.10 |
| 1 x 2 x 3 | 0.99 | 0.15 | 0.74,1.32 | 1.04 | 0.16 | 0.78,1.39 |
| 1 x 2 x 4 | 1.04 | 0.16 | 0.78,1.40 | 1.08 | 0.16 | 0.80,1.45 |
| 1 x 3 x 4 | 1.00 | 0.15 | 0.74,1.33 | 0.93 | 0.14 | 0.70,1.26 |
| 2 x 3 x 4 | 1.44 | 0.42 | 0.80,2.54 | 1.31 | 0.37 | 0.72,2.29 |
| 1 x 2 x 3 x 4 | 0.84 | 0.18 | 0.56,1.29 | 0.85 | 0.18 | 0.56,1.26 |
| **Random Effects** | | | | | | |
| σ^2^ | 1.19 | | | 0.75 | | |
| τ_00_ | 1.13 | | | 0.97 | | |
| ICC | 0.51 | | | 0.44 | | |
| *N* | 114 _CASE_ | | | 114 _CASE_ | | |
| Observations | 912 | | | 912 | | |

*Note.* The regression was fit using non-informative uniform prior assuming a cumulative ordered probit distribution. When calculating R² predictions are treated as continuous variables which is likely invalid for ordinal families for this reason we do not show R².

**Figure S5**

*Marginal Effects Plot for the Interaction between Attitude Towards the Group and Head Cover for Attractiveness*
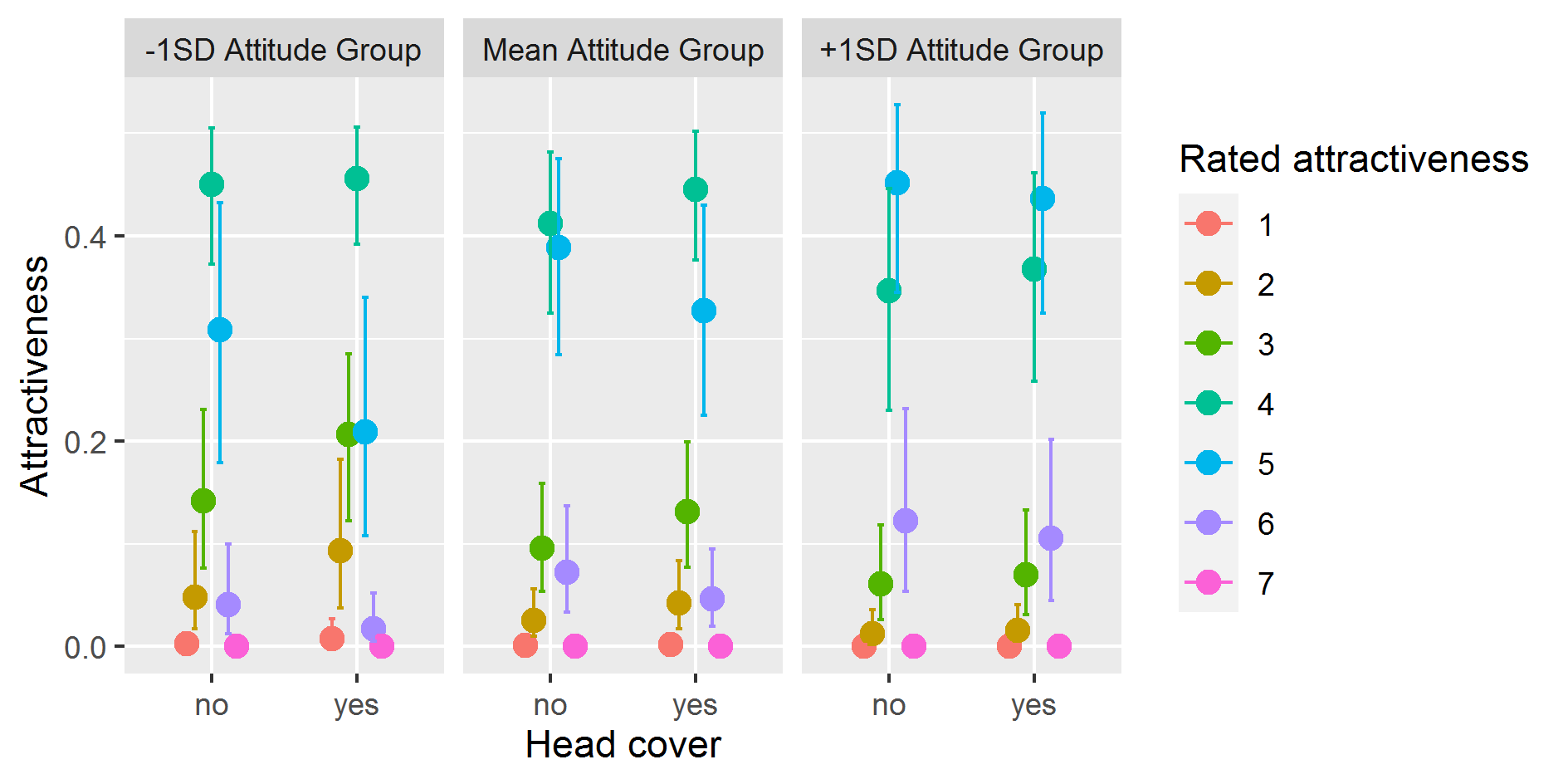


**Figure S6**

*Marginal Effects Plot for the Interaction between Attitude Towards the Group and Head Cover for Liking*

**
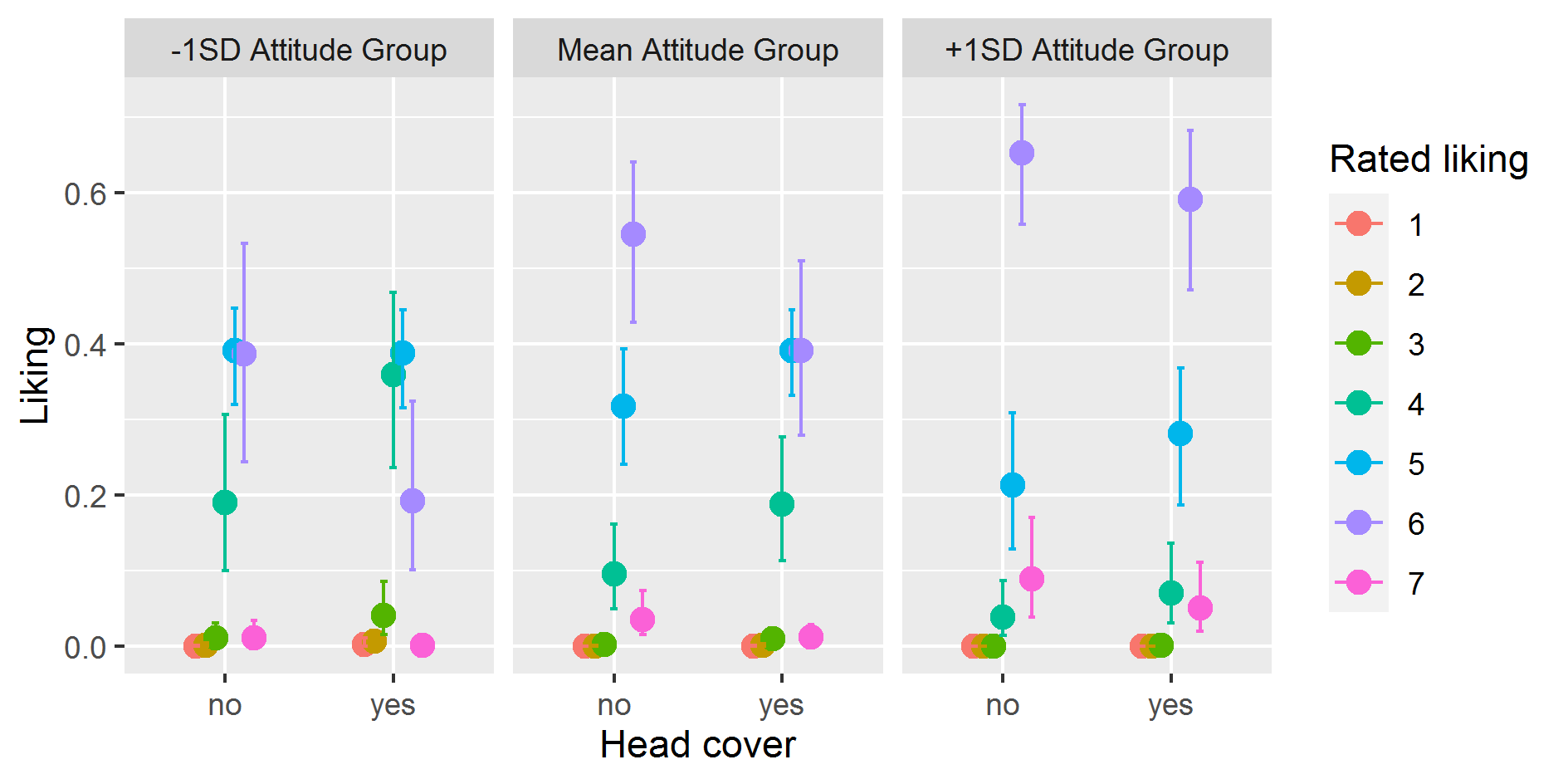
**

**Table S10**

*Results of the Ordinal Regression of Positive and Negative Character on Experimental Factors Testing H2*

|  | **Negative character** | | | **Positive character** | | |
| --- | --- | --- | --- | --- | --- | --- |
| *Predictors* | *Odds Ratios* | *std. Error* | *CI (95%)* | *Odds Ratios* | *std. Error* | *CI (95%)* |
| Attitude group (1) | 0.82 | 0.07 | 0.69,0.98 | 1.51 | 0.12 | 1.27,1.76 |
| Head cover (2) | 1.08 | 0.06 | 0.97,1.20 | 0.82 | 0.05 | 0.73,0.93 |
| Male (3) | 1.12 | 0.06 | 1.00,1.25 | 0.85 | 0.05 | 0.75,0.95 |
| Partner different (4) | 1.00 | 0.06 | 0.90,1.12 | 1.02 | 0.06 | 0.91,1.15 |
| 1 x 2 | 0.88 | 0.04 | 0.81,0.95 | 1.19 | 0.05 | 1.09,1.29 |
| 1 x 3 | 0.98 | 0.04 | 0.90,1.06 | 0.99 | 0.04 | 0.90,1.07 |
| 2 x 3 | 1.04 | 0.08 | 0.89,1.21 | 0.98 | 0.08 | 0.83,1.16 |
| 1 x 4 | 0.99 | 0.04 | 0.91,1.07 | 1.05 | 0.05 | 0.96,1.14 |
| 2 x 4 | 1.00 | 0.08 | 0.86,1.17 | 1.07 | 0.09 | 0.91,1.26 |
| 3 x 4 | 1.03 | 0.08 | 0.88,1.20 | 0.91 | 0.08 | 0.77,1.08 |
| 1 x 2 x 3 | 1.13 | 0.07 | 1.01,1.27 | 0.93 | 0.06 | 0.83,1.05 |
| 1 x 2 x 4 | 1.06 | 0.06 | 0.94,1.19 | 0.98 | 0.06 | 0.87,1.11 |
| 1 x 3 x 4 | 1.06 | 0.06 | 0.95,1.20 | 0.95 | 0.06 | 0.85,1.07 |
| 2 x 3 x 4 | 1.01 | 0.11 | 0.81,1.26 | 0.96 | 0.11 | 0.77,1.22 |
| 1 x 2 x 3 x 4 | 0.85 | 0.07 | 0.72,1.00 | 1.00 | 0.09 | 0.83,1.17 |
| **Random Effects** | | | | | | |
| σ^2^ | 0.79 | | | 0.56 | | |
| τ_00_ | 0.99 | | | 1.09 | | |
| ICC | 0.44 | | | 0.34 | | |
| N | 114 _CASE_ | | | 114 _CASE_ | | |
|  | 8 _question_ | | | 6 _question_ | | |
| Observations | 7296 | | | 5472 | | |

*Note.* The regression was fit using non-informative uniform prior assuming a cumulative ordered probit distribution. When calculating R² predictions are treated as continuous variables which is likely invalid for ordinal families for this reason we do not show R².

**Figure S7**

*Marginal Effects Plot for the Interaction between Attitude Towards the Group and Head Cover for Negative Character*

*
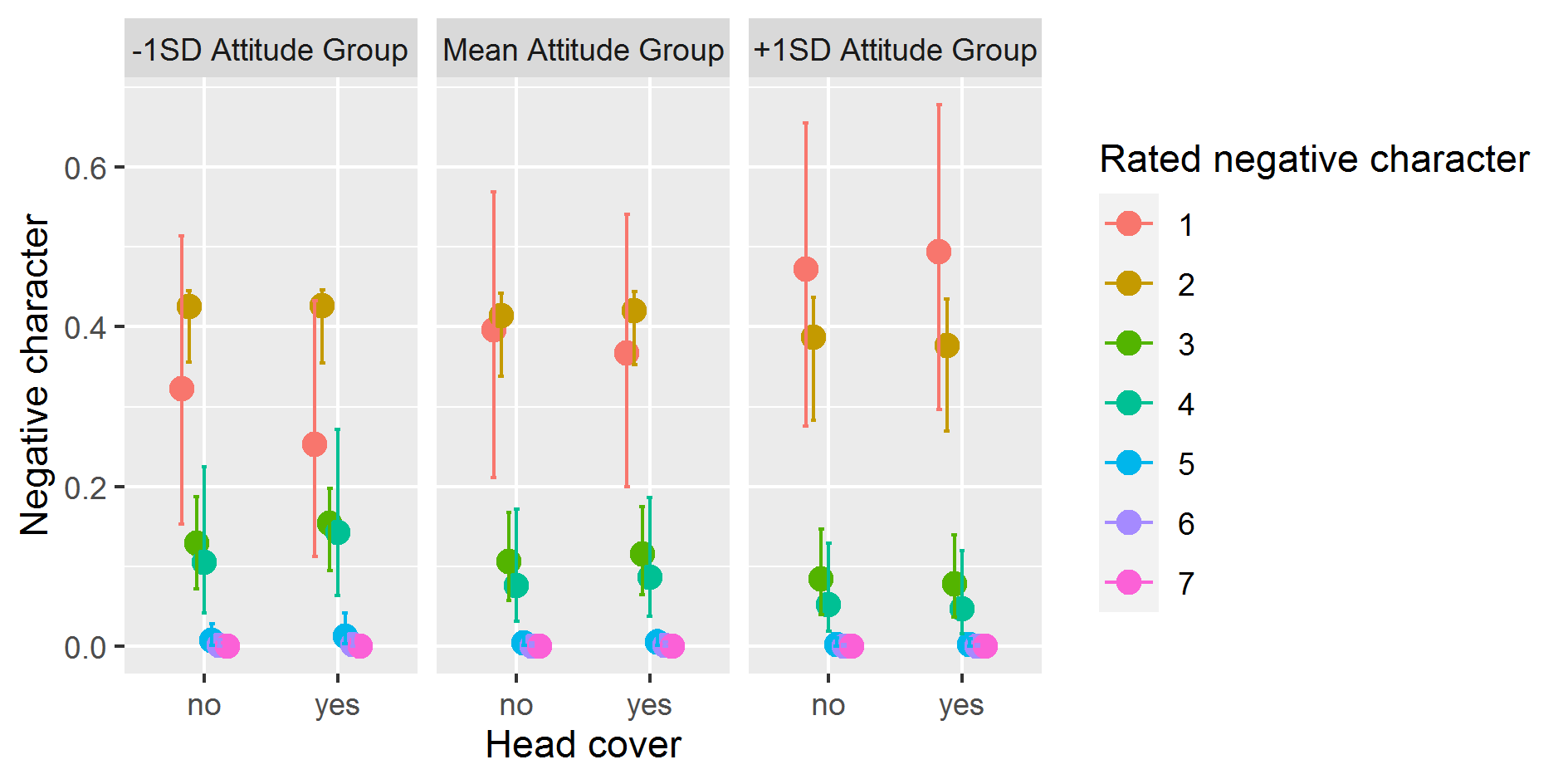
*

**Figure S8**

*Marginal Effects Plot for the Interaction between Attitude Towards the Group and Head Cover for Positive Character*

*
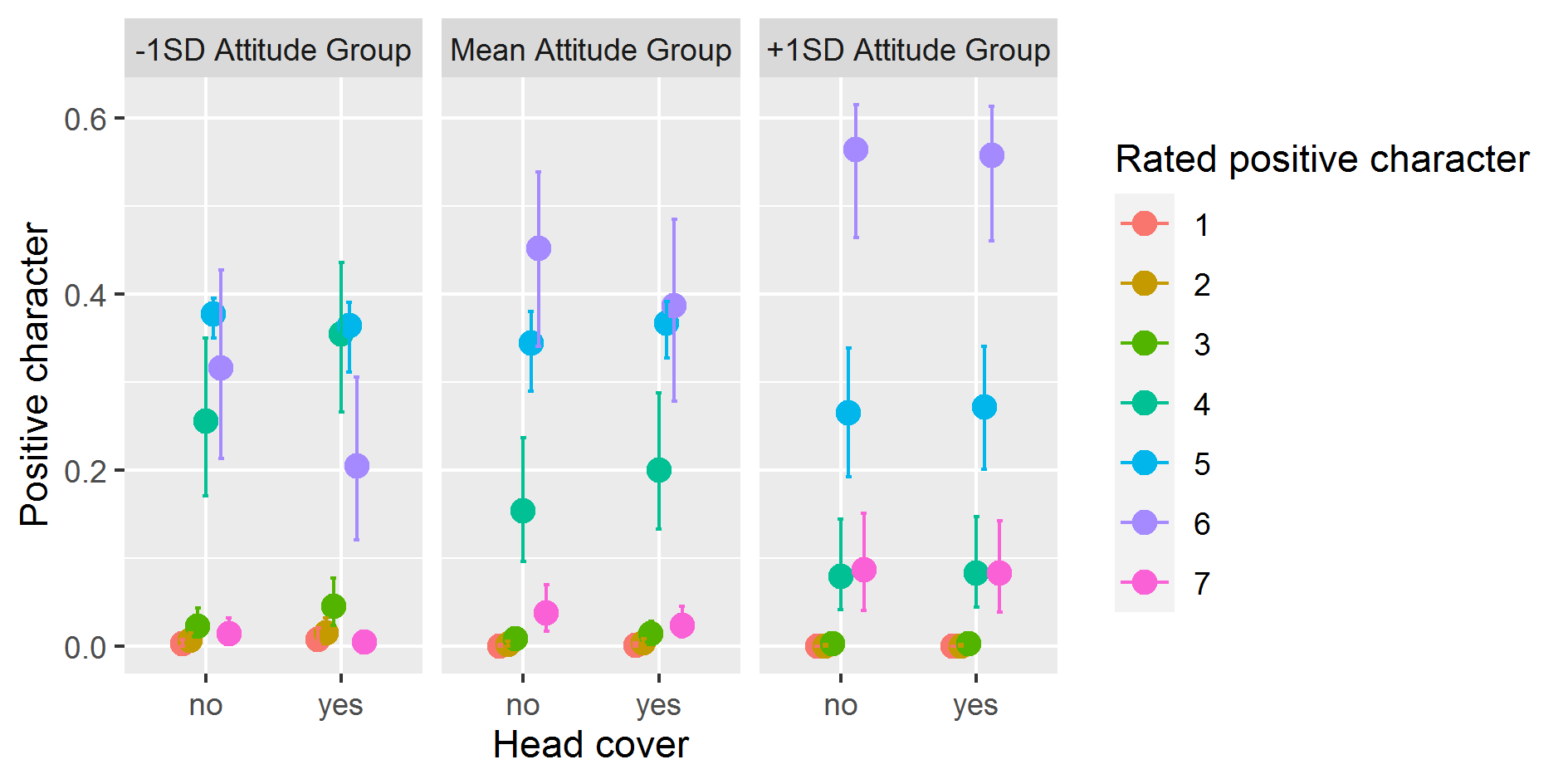
*

Supplement G – Experiment 2 Analyses After Applying Exclusion Criterion

**Table S11**

*Results Linear Regression for the Full Factorial Models for H1*

|  | **Attractiveness** | | | | **Liking** | | | | |
| --- | --- | --- | --- | --- | --- | --- | --- | --- | --- |
| *Predictors* | *B* | *CI* | *β* | *standardized CI* | *B* | *CI* | *β* | *standardized CI* | |
| Int. | 4.30 ^***^ | 3.98; 4.61 | 0.00 | -0.18; 0.18 | 5.38 ^***^ | 5.13; 5.63 | -0.00 | -0.16; 0.16 | |
| Attitude Group (1) | 0.23 | -0.01; 0.48 | 0.25 | 0.07; 0.42 | 0.29 ^**^ | 0.09; 0.48 | 0.38 | 0.22; 0.54 | |
| Headcover (2) | -0.18 | -0.43; 0.08 | -0.09 | -0.13; -0.04 | -0.35 ^**^ | -0.57; -0.12 | -0.11 | -0.15; -0.06 | |
| Male (3) | -0.33 ^*^ | -0.58; -0.07 | -0.15 | -0.19; -0.11 | -0.13 | -0.36; 0.09 | -0.08 | -0.12; -0.03 | |
| Partner different (4) | 0.06 | -0.19; 0.32 | -0.01 | -0.05; 0.03 | -0.07 | -0.30; 0.15 | -0.02 | -0.07; 0.02 | |
| 1 x 2 | 0.15 | -0.05; 0.34 | 0.03 | -0.02; 0.07 | 0.16 | -0.01; 0.33 | 0.06 | 0.02; 0.11 | |
| 1 x 3 | 0.05 | -0.14; 0.25 | -0.02 | -0.06; 0.03 | 0.00 | -0.17; 0.17 | -0.04 | -0.09; 0.00 | |
| 2 x 3 | -0.22 | -0.58; 0.14 | -0.01 | -0.06; 0.03 | 0.01 | -0.31; 0.33 | 0.00 | -0.04; 0.04 | |
| 1 x 4 | -0.02 | -0.21; 0.18 | -0.01 | -0.06; 0.03 | 0.06 | -0.11; 0.24 | 0.03 | -0.02; 0.07 | |
| 2 x 4 | -0.15 | -0.51; 0.21 | 0.00 | -0.04; 0.04 | 0.10 | -0.22; 0.42 | 0.02 | -0.02; 0.07 | |
| 3 x 4 | -0.20 | -0.56; 0.16 | -0.01 | -0.05; 0.04 | -0.10 | -0.42; 0.21 | -0.02 | -0.06; 0.03 | |
| 1 x 2 x 3 | -0.12 | -0.39; 0.16 | -0.05 | -0.09; -0.00 | -0.10 | -0.34; 0.14 | -0.04 | -0.08; 0.01 | |
| 1 x 2 x 4 | 0.03 | -0.24; 0.31 | -0.01 | -0.06; 0.03 | 0.06 | -0.19; 0.30 | 0.00 | -0.04; 0.05 | |
| 1 x 3 x 4 | 0.03 | -0.25; 0.31 | -0.01 | -0.06; 0.03 | -0.03 | -0.28; 0.21 | -0.02 | -0.06; 0.03 | |
| 2 x 3 x 4 | 0.37 | -0.14; 0.88 | 0.03 | -0.01; 0.07 | 0.04 | -0.41; 0.49 | 0.00 | -0.04; 0.05 | |
| 1 x 2 x 3 x 4 | -0.19 | -0.58; 0.20 | -0.02 | -0.06; 0.02 | -0.08 | -0.43; 0.26 | -0.01 | -0.06; 0.03 | |
| **Random Effects** | | | | | | | | |  |
| σ^2^ | 0.68 | | | | 0.53 | | | | |
| τ_00_ | 1.42 _CASE_ | | | | 0.78 _CASE_ | | | | |
| ICC | 0.68 | | | | 0.60 | | | | |
| *N* | 82 _CASE_ | | | | 82 _CASE_ | | | | |
| Observations | 656 | | | | 656 | | | | |
| Marginal *R*^2^ / Conditional *R*^2^ | 0.095 / 0.708 | | | | 0.171 / 0.666 | | | | |
| AIC | 1907.450 | | | | 1719.600 | | | | |
| log-Likelihood | -935.725 | | | | -841.800 | | | | |
| ** p<0.05   ** p<0.01   *** p<0.001* | | | | | | | | |  |

**Table S12**

*Results Linear Regression for the Full Factorial Model for Test of H2*

|  | | | **Negative Character** | | | **Positive Character** | | |
| --- | --- | --- | --- | --- | --- | --- | --- | --- |
| *Predictors* | *B* | *CI* | *β* | *standardized CI* | *B* | *CI* | *β* | *standardized CI* |
| Int. | 2.05 ^***^ | 1.84; 2.25 | 0.00 | -0.20; 0.20 | 5.26 ^***^ | 5.07; 5.45 | 0.00 | -0.17; 0.17 |
| Attitude Group (1) | -0.10 | -0.26; 0.06 | -0.17 | -0.37; 0.03 | 0.28 ^***^ | 0.14; 0.42 | 0.46 | 0.28; 0.63 |
| Headcover (2) | 0.05 | -0.07; 0.17 | 0.03 | 0.00; 0.07 | -0.18 ^**^ | -0.30; -0.05 | -0.07 | -0.10; -0.04 |
| Male (3) | 0.10 | -0.01; 0.22 | 0.06 | 0.03; 0.09 | -0.11 | -0.23; 0.01 | -0.07 | -0.10; -0.04 |
| Partner different (4) | 0.04 | -0.08; 0.16 | 0.00 | -0.03; 0.04 | -0.07 | -0.20; 0.05 | -0.01 | -0.04; 0.02 |
| 1 x 2 | -0.05 | -0.14; 0.04 | -0.00 | -0.04; 0.03 | 0.10 ^*^ | 0.01; 0.19 | 0.04 | 0.01; 0.07 |
| 1 x 3 | -0.05 | -0.14; 0.04 | 0.01 | -0.02; 0.04 | 0.03 | -0.07; 0.12 | -0.02 | -0.05; 0.01 |
| 2 x 3 | 0.04 | -0.13; 0.21 | 0.02 | -0.01; 0.05 | -0.00 | -0.17; 0.17 | -0.02 | -0.06; 0.01 |
| 1 x 4 | -0.02 | -0.11; 0.07 | -0.00 | -0.04; 0.03 | 0.03 | -0.06; 0.13 | 0.01 | -0.02; 0.04 |
| 2 x 4 | -0.04 | -0.21; 0.13 | -0.00 | -0.03; 0.03 | 0.13 | -0.04; 0.31 | 0.01 | -0.02; 0.05 |
| 3 x 4 | -0.04 | -0.21; 0.13 | -0.00 | -0.03; 0.03 | 0.05 | -0.12; 0.22 | -0.01 | -0.04; 0.02 |
| 1 x 2 x 3 | 0.11 | -0.02; 0.24 | 0.03 | -0.00; 0.06 | -0.04 | -0.17; 0.10 | -0.03 | -0.06; -0.00 |
| 1 x 2 x 4 | 0.01 | -0.12; 0.14 | -0.01 | -0.04; 0.02 | 0.02 | -0.11; 0.15 | -0.01 | -0.05; 0.02 |
| 1 x 3 x 4 | 0.05 | -0.08; 0.18 | 0.01 | -0.03; 0.04 | -0.01 | -0.14; 0.13 | -0.02 | -0.05; 0.01 |
| 2 x 3 x 4 | 0.07 | -0.17; 0.31 | 0.01 | -0.02; 0.04 | -0.15 | -0.39; 0.10 | -0.02 | -0.05; 0.01 |
| 1 x 2 x 3 x 4 | -0.07 | -0.25; 0.11 | -0.01 | -0.04; 0.02 | -0.12 | -0.31; 0.06 | -0.02 | -0.05; 0.01 |
| **Random Effects** | | | | | | | | |
| σ^2^ | | | 0.15 | | | 0.15 | | |
| τ_00_ | | | 0.75 _CASE_ | | | 0.57 _CASE_ | | |
| ICC | | | 0.83 | | | 0.79 | | |
| *N* | | | 82 _CASE_ | | | 82 _CASE_ | | |
| Observations | | | 656 | | | 656 | | |
| Marginal *R*^2^ / Conditional *R*^2^ | | | 0.034 / 0.840 | | | 0.221 / 0.834 | | |
| AIC | | | 1004.507 | | | 1005.006 | | |
| log-Likelihood | | | -484.254 | | | -484.503 | | |
| ** p<0.05   ** p<0.01   *** p<0.001* | | | | | | | | |

**Table S13**

*Results of the Ordinal Regression for the Full Factorial Models Testing H1*

|  | **Attractiveness** | | | **Liking** | | |
| --- | --- | --- | --- | --- | --- | --- |
| *Predictors* | *Odds Ratios* | *std. Error* | *CI (95%)* | *Odds Ratios* | *std. Error* | *CI (95%)* |
| Attitude group (1) | 1.47 | 0.26 | 1.03,2.07 | 1.62 | 0.26 | 1.18,2.23 |
| Head cover (2) | 0.80 | 0.14 | 0.57,1.14 | 0.58 | 0.11 | 0.41,0.84 |
| Male (3) | 0.66 | 0.12 | 0.47,0.94 | 0.79 | 0.14 | 0.55,1.15 |
| Partner different (4) | 1.12 | 0.20 | 0.80,1.58 | 0.98 | 0.18 | 0.69,1.40 |
| 1 x 2 | 1.22 | 0.17 | 0.93,1.61 | 1.21 | 0.16 | 0.91,1.59 |
| 1 x 3 | 1.11 | 0.15 | 0.85,1.46 | 0.98 | 0.14 | 0.75,1.31 |
| 2 x 3 | 0.70 | 0.17 | 0.43,1.13 | 0.95 | 0.24 | 0.56,1.54 |
| 1 x 4 | 0.97 | 0.14 | 0.74,1.25 | 1.07 | 0.15 | 0.82,1.41 |
| 2 x 4 | 0.78 | 0.19 | 0.48,1.27 | 1.13 | 0.30 | 0.67,1.82 |
| 3 x 4 | 0.71 | 0.17 | 0.43,1.15 | 0.75 | 0.19 | 0.45,1.23 |
| 1 x 2 x 3 | 0.85 | 0.17 | 0.58,1.25 | 0.90 | 0.17 | 0.61,1.32 |
| 1 x 2 x 4 | 1.07 | 0.21 | 0.74,1.57 | 1.21 | 0.24 | 0.83,1.78 |
| 1 x 3 x 4 | 1.06 | 0.20 | 0.72,1.53 | 0.95 | 0.18 | 0.64,1.40 |
| 2 x 3 x 4 | 1.75 | 0.59 | 0.89,3.57 | 1.18 | 0.42 | 0.60,2.42 |
| 1 x 2 x 3 x 4 | 0.76 | 0.21 | 0.44,1.31 | 0.81 | 0.22 | 0.47,1.42 |
| **Random Effects** | | | | | | |
| σ^2^ | 1.20 | | | 0.77 | | |
| τ_00_ | 1.08 | | | 0.81 | | |
| ICC | 0.53 | | | 0.49 | | |
| *N* | 82 _CASE_ | | | 82 _CASE_ | | |
| Observations | 656 | | | 656 | | |

*Note.* The regression was fit using non-informative uniform prior assuming a cumulative ordered probit distribution. When calculating R² predictions are treated as continuous variables which is likely invalid for ordinal families for this reason we do not show R².

**Table S14**

*Results of the Ordinal Regression for the Full Factorial Models Testing H2*

|  | **Negative character** | | | **Positive character** | | |
| --- | --- | --- | --- | --- | --- | --- |
| *Predictors* | *Odds Ratios* | *std. Error* | *CI (95%)* | *Odds Ratios* | *std. Error* | *CI (95%)* |
| Attitude group (1) | 0.91 | 0.10 | 0.73,1.16 | 1.48 | 0.15 | 1.20,1.81 |
| Head cover (2) | 1.05 | 0.07 | 0.94,1.20 | 0.81 | 0.06 | 0.70,0.92 |
| Male (3) | 1.11 | 0.07 | 0.98,1.26 | 0.84 | 0.06 | 0.73,0.96 |
| Partner different (4) | 1.02 | 0.07 | 0.89,1.16 | 0.92 | 0.06 | 0.80,1.06 |
| 1 x 2 | 0.91 | 0.05 | 0.82,1.01 | 1.13 | 0.06 | 1.01,1.26 |
| 1 x 3 | 0.92 | 0.05 | 0.83,1.01 | 1.03 | 0.06 | 0.92,1.15 |
| 2 x 3 | 1.11 | 0.10 | 0.93,1.34 | 1.01 | 0.10 | 0.83,1.23 |
| 1 x 4 | 0.96 | 0.05 | 0.86,1.07 | 1.02 | 0.06 | 0.92,1.14 |
| 2 x 4 | 0.98 | 0.09 | 0.81,1.17 | 1.17 | 0.12 | 0.96,1.44 |
| 3 x 4 | 1.00 | 0.09 | 0.83,1.19 | 1.05 | 0.10 | 0.87,1.29 |
| 1 x 2 x 3 | 1.19 | 0.08 | 1.03,1.38 | 0.96 | 0.07 | 0.82,1.12 |
| 1 x 2 x 4 | 1.05 | 0.08 | 0.90,1.21 | 1.05 | 0.08 | 0.91,1.23 |
| 1 x 3 x 4 | 1.09 | 0.08 | 0.95,1.26 | 1.01 | 0.08 | 0.87,1.18 |
| 2 x 3 x 4 | 1.01 | 0.13 | 0.78,1.29 | 0.82 | 0.11 | 0.62,1.07 |
| 1 x 2 x 3 x 4 | 0.88 | 0.09 | 0.72,1.07 | 0.83 | 0.09 | 0.67,1.03 |
| **Random Effects** | | | | | | |
| σ^2^ | 0.74 | | | 0.65 | | |
| τ_00_ | 0.95 | | | 0.91 | | |
| ICC | 0.44 | | | 0.42 | | |
| *N* | 82 _CASE_ | | | 82 _CASE_ | | |
|  | 8 _question_ | | | 6 _question_ | | |
| Observations | 5248 | | | 3936 | | |

*Note.* The regression was fit using non-informative uniform prior assuming a cumulative ordered probit distribution. When calculating R² predictions are treated as continuous variables which is likely invalid for ordinal families for this reason we do not show R².
